# Structural and functional analysis of Dickkopf 4 (Dkk4): New insights into Dkk evolution and regulation of Wnt signaling by Dkk and Kremen proteins

**DOI:** 10.1074/jbc.RA118.002918

**Published:** 2018-06-20

**Authors:** Saleha Patel, Alice M. Barkell, Deepti Gupta, Sarah L. Strong, Shaun Bruton, Frederick W. Muskett, Philip W. Addis, Philip S. Renshaw, Patrick M. Slocombe, Carl Doyle, Alison Clargo, Richard J. Taylor, Christine E. Prosser, Alistair J. Henry, Martyn K. Robinson, Lorna C. Waters, Gill Holdsworth, Mark D. Carr

**Affiliations:** From the ‡ Leicester Institute of Structural and Chemical Biology and; the § Department of Molecular and Cell Biology, University of Leicester, Lancaster Road, Leicester LE1 7HB, United Kingdom and; ¶ UCB, 208 Bath Road, Slough SL1 3WE, United Kingdom

**Keywords:** cooperativity, nuclear magnetic resonance (NMR), Wnt signaling, cell surface receptor, structural biology, protein structure, protein-protein interaction, Dkk4, Kremen1, LRP6

## Abstract

Dickkopf (Dkk) family proteins are important regulators of Wnt signaling pathways, which play key roles in many essential biological processes. Here, we report the first detailed structural and dynamics study of a full-length mature Dkk protein (Dkk4, residues 19–224), including determination of the first atomic-resolution structure for the N-terminal cysteine-rich domain (CRD1) conserved among Dkk proteins. We discovered that CRD1 has significant structural homology to the Dkk C-terminal cysteine-rich domain (CRD2), pointing to multiple gene duplication events during Dkk family evolution. We also show that Dkk4 consists of two independent folded domains (CRD1 and CRD2) joined by a highly flexible, nonstructured linker. Similarly, the N-terminal region preceding CRD1 and containing a highly conserved N*X*I(R/K) sequence motif was shown to be dynamic and highly flexible. We demonstrate that Dkk4 CRD2 mediates high-affinity binding to both the E1E2 region of low-density lipoprotein receptor–related protein 6 (LRP6 E1E2) and the Kremen1 (Krm1) extracellular domain. In contrast, the N-terminal region alone bound with only moderate affinity to LRP6 E1E2, consistent with binding via the conserved N*X*I(R/K) motif, but did not interact with Krm proteins. We also confirmed that Dkk and Krm family proteins function synergistically to inhibit Wnt signaling. Insights provided by our integrated structural, dynamics, interaction, and functional studies have allowed us to refine the model of synergistic regulation of Wnt signaling by Dkk proteins. Our results indicate the potential for the formation of a diverse range of ternary complexes comprising Dkk, Krm, and LRP5/6 proteins, allowing fine-tuning of Wnt-dependent signaling.

## Introduction

**Figure 1.**
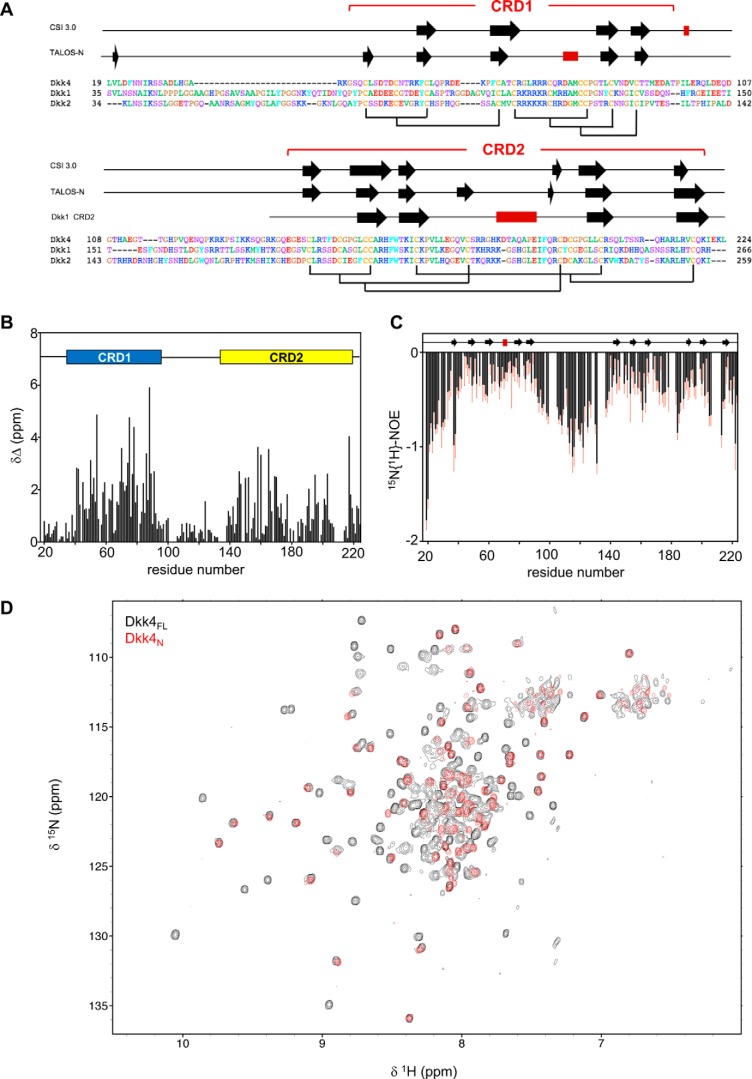
**Structural properties and features of full-length Dkk4.** *A*, sequence alignment of mature human Dkk proteins obtained using Clustal O, with residues numbered from the N-terminal methionine. Indicated *above* are the regular secondary structure elements predicted from backbone NMR assignments obtained for Dkk4_FL_ using CSI 3.0 and TALOS-N, with β-strands and α-helices represented by *black arrows* and *red rectangles*, respectively. The secondary structure observed for Dkk1 CRD2 in the crystal structure bound to LRP6 E3E4 is also shown (PDB code 3S2K). The expected disulfide bond pattern for Dkk4_FL_ is shown *below* the sequences, and the locations of CRD1 and CRD2 are also indicated. *B*, the absolute combined ^1^H^N^ and ^15^N^H^ secondary chemical shifts for Dkk4_FL_ determined by calculating the difference between the observed and predicted random coil chemical shift values for each residue. A schematic of the domain architecture is shown *above*, indicating the positions of CRD1 and CRD2. *C*, heteronuclear ^15^N{1H}-NOE values determined for Dkk4_FL_. *Error bars*, shown in *red*, indicate an estimate of the error on the measurement. The regular secondary structure predicted by TALOS-N is also indicated above. *D*, an overlay of ^15^N/^1^H TROSY spectra acquired for Dkk4_FL_ (*black*) and Dkk4_N_ (*red*).

Wnt signaling pathways are regulated through a complex network of effector molecules that act as activators and inhibitors. These modulate a great variety of developmental processes, including cellular differentiation, cell fate determination, organogenesis, mitogenic stimulation, and stem cell maintenance (1). The Dickkopf (Dkk)^7^ family of cysteine-rich secretory proteins are highly conserved inhibitors of the canonical Wnt signaling pathway. Four Dkk proteins exist in humans, and all contain two cysteine-rich domains (CRDs), designated CRD1 and CRD2, each of which contains five disulfide bonds (Fig. 1*A*). Dkk1, Dkk2, and Dkk4, but not Dkk3, have been identified as potent inhibitors of Wnt signaling and bind to the Wnt co-receptors LRP5/6 (2–4). Dkk1 and Dkk2 have been shown to bind to both the isolated LRP6 β-propeller domains 1 and 2 (E1E2) and β-propeller domains 3 and 4 (E3E4), effectively competing with Wnts that are known to bind specifically to either the LRP6 E1 or E3 regions (3–7). Furthermore, CRD2 has been found to be crucial for Wnt signaling inhibition, with the isolated Dkk1 and Dkk2 CRD2 inhibiting Wnt8 signaling (8). It is now well established that the Dkk1 CRD2 binds directly to LRP6, and several crystal structures of Dkk1 CRD2 in complex with LRP6 E3E4 have been published (9, 10). Additional insights into the mechanism by which Dkk proteins may inhibit Wnt signaling come from reports that the N-terminal N*X*I(R/K) sequence motif found in the region preceding CRD1 interacts with LRP6 E1 (11).

To add further complexity, the Kremen (Krm) family of receptors, which consists of the evolutionarily conserved homologues Krm1 and Krm2, have also been shown to tightly bind to both Dkk1 and Dkk2 and together can form a ternary complex with LRP6 (12). Dkk1 alone has been shown to inhibit Wnt signaling through interactions with LRP6; however, the presence of Krm has been reported to significantly increase the inhibitory potency of Dkk1 (12). The Krm family extracellular region (ECD) contains individual kringle (Krg), WSC, and CUB domains, which all appear to be required for functional interaction with Dkks (12). The structure of the Krm1 ECD has recently been reported, together with a low-resolution crystal structure of the Krm1 ECD in complex with Dkk1 CRD2 and LRP6 E3E4 (13).

Dkk4 is the smallest member of the Dkk family and the least studied. However, it is emerging as an important regulator of Wnt signaling and has recently been implicated in colon carcinogenesis and hepatocellular carcinoma (14, 15). Here, we report the first detailed structural and dynamics study of a full-length mature Dkk protein, as typified by Dkk4 (residues 19–224), including the first atomic-resolution structure obtained for the CRD1 domain of a Dkk family protein. This unexpectedly revealed that the N- and C-terminal cysteine-rich domains of Dkk proteins (CRD1 and CRD2) share significant structural homology, which has important implications for the evolution of Dkk family proteins. We also thoroughly characterized the interactions of Dkk4 with its key functional partners LRP6 and Krm1/2, using a combination of pulldown, biosensor, and NMR experiments, to conclusively show that it is the CRD2 region of Dkk4 that mediates high-affinity binding to LRP6 E1E2 and Krm1 ECD. In addition, we confirm the functional synergy between Dkk and Krm proteins in regulating Wnt-mediated signaling. The detailed structural, dynamics, interaction, and functional data obtained for Dkk4, together with reported work on Dkk1 and Dkk2, have allowed the development of a highly informative proposed model for synergistic regulation of Wnt signaling by Dkk and Krm family proteins. Importantly, this highlights the potential for very fine-tuning of the inhibition of Wnt signaling via a range of ternary LRP–Dkk–Krm complexes.

## Results

### Initial characterization of recombinant human Dkk proteins

Recombinant full-length mature Dkk2 (Dkk2_FL_) and Dkk4 (Dkk4_FL_) with their N-terminal signal sequence removed (residues 38–259 and 19–224, respectively) were expressed in *Escherichia coli* as insoluble inclusion bodies, which were subsequently isolated, the proteins individually refolded and purified to homogeneity. Mass spectrometry analysis under reduced and nonreduced conditions confirmed that proteins of the expected molecular weights were obtained, with all of the conserved cysteines involved in disulfide bonds. CD analysis of Dkk4_FL_ showed that it was stable up to about 40 °C (Fig. S1*A*). 2D ^15^N/^1^H TROSY spectra of uniformly ^15^N-labeled Dkk4_FL_ and Dkk2_FL_ contained a large number of well-dispersed signals, together with a region containing many relatively intense and overlapped peaks, which indicates the presence of both structured domains and nonstructured, relatively mobile regions (Fig. S1, *B* and *E*).

We also confirmed the ability of refolded Dkk2_FL_ and Dkk4_FL_ to bind LRP6. The refolded proteins were added to cells expressing full-length LRP6, and FACS analysis demonstrated the expected binding of both Dkks to LRP6 (Fig. S1, *C* and *F*). In addition, refolded Dkk4_FL_ showed concentration-dependent inhibition of Wnt signaling in HEK293-Luc cells stimulated with Wnt3a conditioned medium (Fig. S1*D*). The behavior of refolded Dkk4_FL_ was very similar to that observed when an expression vector for Dkk4 was transfected into the same reporter cells (Fig. S2). In these assays, Dkk4 was able to inhibit Wnts that bind LRP6 E1 and E3 (Wnt1 and Wnt3a, respectively) but appeared to be less potent than Dkk1, as observed previously (16). Overall, our data show that refolded Dkk4_FL_ and Dkk2_FL_ have the expected biological activity, confirming that both proteins were correctly refolded.

### Structure and dynamics of full-length Dkk4

We have previously reported comprehensive sequence-specific backbone resonance assignments for Dkk4_FL_ at pH 5.5 (17); however, to allow NMR studies of Dkk4 binding to several functional partners (LRP6 and Krm1), backbone assignments were required to be extended to pH 6.5. 2D and 3D NMR spectra acquired for Dkk4_FL_ at pH 6.5 and 5.5 showed relatively few changes, which is reflected in very similar backbone resonance assignments obtained under both conditions. Backbone assignments were made for 179 of the 207 residues in Dkk4_FL_ (86.5%) at pH 6.5, and analysis of the backbone amide chemical shifts (^15^N and ^1^H) revealed that many of the signals in CRD1 and CRD2 were significantly shifted from random coil values (Fig. 1*B*), indicating that these regions are largely structured. The range of ^15^N{1H}-NOE values determined for CRD1 and CRD2 are also consistent with structured domains (Fig. 1*C*), but several regions of CRD2 show heteronuclear NOEs indicative of highly dynamic regions, such as the loop connecting the two β-sheets that is involved in Dkk1 binding to LRP6 E3 (18, 19).

The N-terminal residues (residues 19–39) of Dkk4, together with the linker region between CRD1 and CRD2 (residues 98–140), show close to random coil chemical shift values for backbone amide signals and are characterized by ^15^N{1H}-NOEs expected for highly mobile, nonstructured regions of proteins. Similarly, the range of predicted order parameters (S^2^ of 0.35–0.7) derived from the backbone NMR assignments using TALOS-N indicates that residues 19–40 and 98–139 and the five most carboxyl-terminal residues of the protein (residues 220–224) are dynamic and highly flexible (20).

Collectively, the NMR data obtained for Dkk4_FL_ reveal that the intact protein consists of two structured domains (CRD1 and CRD2) joined by a highly flexible linker (residues 98–140) and a similarly highly dynamic and highly flexible N-terminal region (residues 19–39). Interestingly, this dynamic and highly flexible N-terminal region contains the conserved N*X*I(R/K) sequence motif, which has been shown to mediate the binding of short peptides to LRP6 (11).

Both CSI 3.0 and TALOS-N analysis of the backbone NMR assignments identified similar regions of secondary structure in Dkk4_FL_ (Fig. 1*A*) (20, 21). The secondary structure predicted for CRD2 is similar to that observed in the published crystal structure determined for Dkk1 CRD2 in complex with LRP6 E3E4 and the solution structure of Dkk2 CRD2 (9, 22). The positions of the β-strands appear well conserved between Dkk1, Dkk2, and Dkk4 CRD2; however, the α-helix formed by residues 227–233 of Dkk1 CRD2 bound to LRP6 E3E4 is absent in free Dkk4. This helix was also not observed in the solution structure reported for the isolated Dkk2 CRD2 (22). Given that the sequence of this region is essentially identical in Dkk1 and Dkk2, it seems likely that this helix is formed upon binding to LRP6. In contrast, the sequence of this region has diverged in Dkk4, making it difficult to predict whether the equivalent helix in Dkk4 would be present in the LRP6-bound state. Overall, the NMR data obtained for CRD2 in full-length mature Dkk4 indicate that this domain has a structure very similar to that determined for the isolated domain from Dkk1 and Dkk2. Given the conservation between these family members, we believe that the structural features and dynamic behavior observed for mature full-length Dkk4 are very likely to be mimicked by Dkk1 and Dkk2.

### Solution structure of Dkk4 CRD1

The secondary structure predicted for Dkk4 CRD1 from the assigned backbone NMR signals suggests the presence of a number of short β-strands linked by loops (Fig. 1*A*). Although several structures of the CRD2 region from Dkk1 and Dkk2 have been published, to date no tertiary structure of the CRD1 region from a Dkk protein has been reported. We therefore sought to determine the solution structure of the entire N-terminal region of Dkk4 (Dkk4_N_), including CRD1. Dkk4_N_ (residues 19–97) was expressed and characterized using an approach similar to that described above for Dkk4_FL_.

Dkk4_N_ gives rise to relatively well-resolved NMR spectra, as illustrated by the ^15^N/^1^H TROSY spectrum shown in Fig. 1*D*. Comparison of the NMR spectra obtained for Dkk4_FL_ and Dkk4_N_ reveals very few significant chemical shift differences between equivalent residues in the full-length protein and isolated N-terminal region of Dkk4, as exemplified in the overlay of the ^15^N/^1^H TROSY spectra shown in Fig. 1*D*, where the majority of peaks arising from the N-terminal region of Dkk4 are closely overlapped in both spectra. Significant chemical shift changes were only observed for the five residues at the C terminus of Dkk4_N_ and for some residues located in the C-terminal linker-His_6_ tag. This indicates that there are no interactions between the two cysteine-rich domains in the intact protein. We obtained comprehensive sequence-specific backbone and side-chain resonance assignments for Dkk4_N_, together with a substantial number of structural constraints from 2D and 3D NOE-based spectra. For example, backbone amide signals (^15^N and ^1^H) were assigned for all nonproline residues except Leu^19^, Ser^29^, His^33^, Arg^36^, Lys^37^, and Asp^44^ (92%) and for all Cα and Cβ apart from Ser^28^, Ser^29^, His^33^, and Arg^36^ (95%). The ^15^N, ^13^C, and ^1^H resonance assignments obtained for Dkk4_N_ have been deposited at the BioMagResBank database (accession number 34146).

The CANDID protocol (23) proved very effective at determining assignments for the NOEs identified in 2D and 3D NOESY-based spectra of Dkk4_N_, with assignments obtained for 95% (1946 of 2042) of the NOE peaks, which produced 606 nonredundant ^1^H to ^1^H upper distance limits. Subsequently, several cycles of simulated annealing combined with redundant dihedral angle constraints (REDAC) were used to produce the final converged Dkk4_N_ structures. The final family of Dkk4_N_ structures was determined using a total of 750 NMR-derived structural constraints (an average of 11.7 per residue for the well-defined core region corresponding to residues 38–97), including 606 NOE-based upper distance limits, 90 backbone torsion angle constraints, 24 hydrogen bond constraints, and 30 disulfide bond constraints. Following the final round of CYANA calculations, 70 satisfactorily converged structures were obtained from 100 random starting structures. The converged structures contain no distance or van der Waals violation greater than 0.5 Å and no dihedral angle violations greater than 5°, with an average value for the CYANA target function of 0.11 ± 0.04 Å^2^. The NMR constraints and structural statistics for Dkk4_N_ are summarized in Table S1. The family of converged Dkk4_N_ structures, together with the NMR constraints, have been deposited in the Protein Data Bank (accession code 5O57).

**Figure 2.**
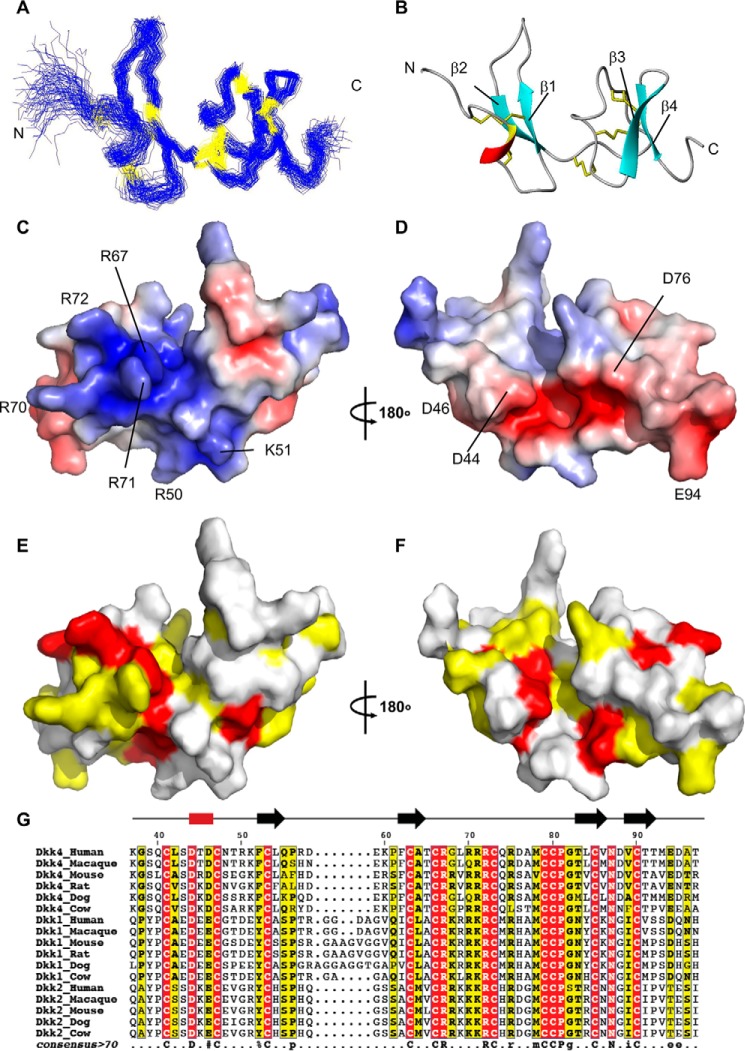
**Structural properties and features of Dkk4 CRD1.** *A*, a best fit superposition of the protein backbone for the family of 70 converged structures (best fit for residues 44–47 and 50–96) obtained for the structured CRD1 region of Dkk4_N_ (residues 37–97). *B*, a *ribbon representation* of the backbone topology of Dkk4 CRD1 in the same orientation as *A*. The locations of the disulfide bonds are shown in *yellow. C* and *D*, *surface views* of Dkk4 CRD1 *colored* according to electrostatic potential, with areas of significant negative charge shown in *red*, significant positive charge in *blue*, and neutral in *white. E* and *F*, *surface views* of Dkk4 CRD1, with residues highlighted on the basis of sequence conservation, with residues that are identical across all the representative mammalian Dkk1, -2, and -4 species shown in *red* and those that are partially conserved in over 70% of the sequences shown in *yellow*. The structures in *D* and *F* are shown in the same orientation as *B*, whereas *C* and *E* have been rotated by 180° about the *y* axis. *G*, multiple-sequence alignment of the structured CRD1 from Dkk1, -2, and -4 (human, macaque, mouse, rat, dog, and cow). Residues with completely conserved sequence identity are highlighted in *red*, and those that have conserved sequence identity in over 70% of the sequences are highlighted in *yellow*. The consensus sequence is shown *below*. Amino acids with completely conserved sequence identity are shown in *uppercase*; those with conserved sequence identity in over 70% of the sequences are shown in *lowercase*. Similar residues are grouped as follows: AVILM, FYW, KRH, DE, STNQ, PG, and C. %, Phe or Tyr; #, any one of Asn, Asp, Gln, or Glu.

The solution structure of CRD1 within Dkk4_N_ is determined to relatively high precision (an average of 11.7 constraints per residue for the well-defined region corresponding to residues 38–97), which is clearly evident from the superposition of the protein backbone shown for the family of converged structures (Fig. 2*A*). This is also reflected in low root mean square deviation (RMSD) values to the mean structure for both the backbone and all heavy atoms (0.90 ± 0.23 and 1.40 ± 0.22 Å, respectively) of residues forming the well-defined core region (residues 44–47 and 50–96).

The NMR data clearly indicate that the first 19 N-terminal residues of Dkk4_N_ (residues 19–37), which includes the largely conserved N*X*I(R/K) motif, are dynamic and highly flexible in solution (Fig. 1). The well-defined structured CRD1 region of Dkk4_N_ contains a single turn of 3_10_ helix (Asp^44^–Asp^46^) and two small antiparallel β-sheets (β-sheet 1: β-strand 1 (Phe^52^–Leu^54^) and β-strand 2 (Phe^62^–Ala^64^); β-sheet 2: β-strand 3 (Thr^83^–Val^86^) and β-strand 4 (Val^89^–Thr^92^)), which are linked by well-defined loops (Fig. 2*B*). The NMR data also suggest that short regions of both the loop connecting β-sheets 1 and 2 (Asp^76^–Met^78^) and the C-terminal region (residues Met^93^–Ala^96^) have the propensity to adopt a helical conformation. The domain contains five disulfide bonds: C41–C53 (C1–C3), C47–C63 (C2–C4), C66–C80 (C5–C8), C73–C85 (C6–C9), and C79–C90 (C7–C10), which, together with a network of hydrogen bonds (Asp^46^ NH–Ser^43^ C′, Cys^47^ NH–Asp^44^ C′, Phe^62^ NH–Leu^54^ C′, Ala^64^ NH–Phe^52^ C′, Arg^71^ NH–Gly^68^ C′, Cys^73^ NH–Asp^88^ C′, Leu^84^ NH–Thr^91^ C′, Val^86^ NH–Val^89^ C′, Val^89^ NH–Val^86^ C′, Cys^90^ NH–Arg^71^ C′, Thr^91^ NH–Leu^84^ C′, Ala^96^ NH–Thr^92^ C′) and a potential salt bridge (Arg^72^–Asp^88^), play a major role in stabilizing the tertiary structure. The long central loop (18 residues), which links the N- and C-subdomains of CRD1, is tethered to the C-terminal β-sheet by two disulfide bonds. The two ends of this loop are also tethered together by a third disulfide bond. Interestingly, comparison of the backbone topology of the N- and C-subdomains of CRD1 shows that both the secondary and tertiary structures and the locations of the C41–C53, C47–C63 and the C73–C85, C79–C90 disulfides are conserved between the two subdomains. The structural similarity is reflected in a backbone RMSD of 1.2 Å between the two subdomains (residues Gln^40^–Gln^55^ and Pro^61^–Thr^65^ of the N-subdomain and Arg^70^–Thr^92^ of the C-subdomain) (PDBeFold (24)).

### Potential functional sites in Dkk4 CRD1

Dkk4 CRD1 has a highly charged surface with large acidic and basic regions. One face of the domain has a large positively charged patch that extends over halfway around the molecule. The patch covers a significant part of the C-subdomain as well as extending into a groove between the two subdomains (Fig. 2*C*). Of particular note are three sequential solvent-exposed arginine residues (Arg^70^, Arg^71^, and Arg^72^), located to one side of the groove. The side chains of these arginines appear to extend from the positive patch like the three prongs of a plug. The opposite face of CRD1 contains a negatively charged cleft, formed by residues in the 3_10_ helix, the N-terminal ends of β-strands 1 and 3, and the joining loops (Fig. 2*D*). The C-terminal end of the cleft joins with a negative patch that extends across both sides of the C-terminal loop and onto the adjacent β-sheets. It is likely that these striking surface features will form major parts of the binding sites for currently unidentified functional partners.

Dkk genes have been identified in vertebrate and some invertebrate phyla. Interestingly, whereas Dkk1, Dkk2 and Dkk3 have been identified in fish, birds, reptiles, amphibians, and mammals, Dkk4 appears to only be present in mammals and reptiles. The amino acid sequence of Dkk4_N_ is highly conserved across the different known mammalian and reptilian species (Fig. S3). Although there are only very short regions of the N-terminal unstructured region and some of the loops linking the regions of regular secondary structure that show any significant variation between the representative species, the N*X*I(R/K) motif does not appear to be conserved in turtle Dkk4 (Fig. S3*A*).

Comparison of the amino acid sequences of a representative set of mammalian Dkk1, Dkk2, and Dkk4 species shows that the CRD1 sequence is well conserved across these functionally related Dkk proteins, suggesting that the topology of the CRD1 domain will be maintained among these Dkk family members (Fig. 2, *E–G*). The least conserved regions are the loops on either side of β-strand 1. The previously described positive patch of residues is seen across the Dkks, with the presence of basic residues at the equivalent locations to Arg^67^, Arg^70^, Arg^71^, and Arg^72^ conserved across the vast majority of sequences. Interestingly, in almost all of the Dkk1 and Dkk2 sequences, there are two basic residues located between Arg^67^ and Arg^70^, resulting in a stretch of six consecutive basic residues, which are flanked at each end by a cysteine. Residues Arg^50^ and Lys^51^ of Dkk4 CRD1 are also located in the positive patch.

Only two of the four acidic residues (Asp^44^ and Asp^46^) located in and around the negative cleft are present across all of the Dkks. The presence of an acidic residue at Glu^94^ is conserved in Dkk4 and Dkk1 but not Dkk2. Asp^76^, which is not well maintained in the other Dkk4 sequences, is conserved in Dkk2 but is substituted for a histidine in the majority of Dkk1 species. These differences in the amino acids present at the potential binding sites for functional partners suggest that subtle differences are expected to exist in the functions of the Dkk family members.

### CRD1 shares structural homology with CRD2

**Figure 3.**
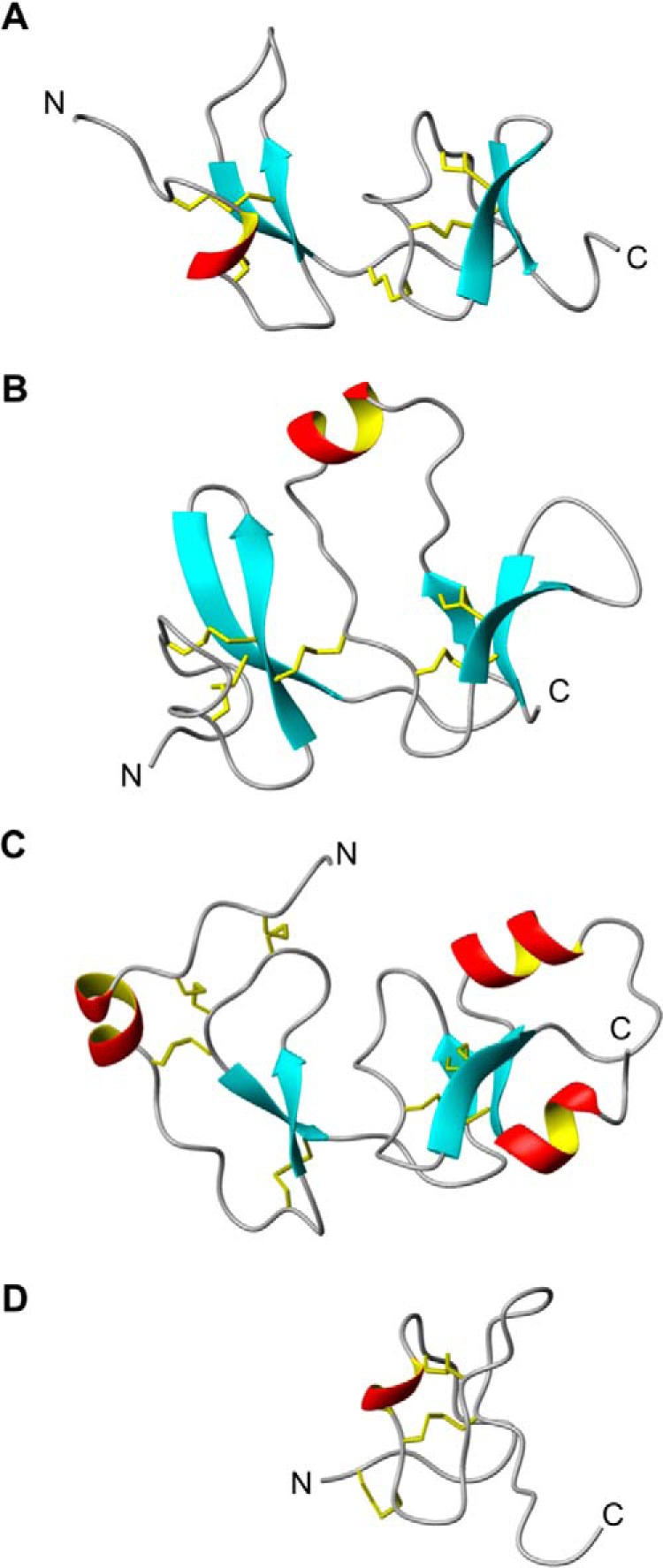
**Structural homologues of Dkk4 CRD1.** *A–D*, *equivalent views* of *ribbon representations* of the backbone structures of Dkk4 CRD1, Dkk1 CRD2, IGFBP4_N_, and the ICK domain of Jingzhaotoxin XI, respectively. The locations of the disulfides are shown in *yellow*.

Comparison of the backbone topology of Dkk4 CRD1 with other known folds in the PDB identified 45 structural neighbors (25). The top 14 hits were all colipase folds (26, 27). Surprisingly, given the low level of amino acid sequence homology (<30%), two of the closest structural homologues found were CRD2 of Dkk1 and Dkk2 (13, 22). A comparison of Dkk4 CRD1 and Dkk1 CRD2 shows that they adopt similar secondary and tertiary structures (Fig. 3, *A* and *B*), which is reflected in a backbone RMSD of 2.1 Å (residues Gln^40^–Arg^57^, Glu^59^–Arg^75^, and Met^78^–Met^93^ of Dkk4 and residues Val^188^–Lys^222^, Tyr^238^–Ile^247^, and His^261^–His^266^ of Dkk1 (13). The most striking difference between the Dkk4 CRD1 and Dkk1 CRD2 is the loop that links the two β-sheets. This region of Dkk1 CRD2 is significantly longer (31 residues) and contains a central helix, which is involved in binding to LRP6 and Kremen. The locations of four of the five disulfides are conserved between CRD1 and CRD2. In CRD1 the C66–C80 disulfide tethers the two ends of the long loop together, whereas in CRD2, it is replaced by one that joins the loop to the first β-sheet (Fig. 3*B*).

Dkk4 CRD1 also shows structural homology to the N-terminal domain of insulin-like growth factor-binding protein-4 (IGFBP-4_N_) (Fig. 3*C*), which is reflected in a backbone RMSD of 4.2 Å (residues Ser^39^–Leu^42^, Asp^46^–Arg^57^, Pro^61^–Gln^74^, and Met^78^–Thr^97^ of Dkk4 and residues Arg^16^–Pro^19^, Val^21^–Cys^32^, Cys^34^–Gly^47^, Arg^52^–Pro^61^, and Gly^77^–Glu^86^ of IGFBP-4 (28). The locations of the two C-terminal disulfide bonds are conserved between Dkk4 CRD1 and IGFBP-4. Interestingly, the C-terminal cysteine-rich domain of IGFBP-4 has been shown to directly interact with both the Wnt receptor Frizzled 8 and LRP6 and inhibit their binding to Wnt3a (29).

The C-subdomain of Dkk4 CRD1 also shows significant structural homology to a number of toxins from the inhibitor cysteine knot (ICK) family of peptides. ICK peptides are found in animals, plants, and fungi and have diverse roles, including functioning as invertebrate toxins, plant protease inhibitors, and mammalian neuropeptides (30). For example, Dkk4 CRD1 shows structural homology to the spider protein Jingzhaotoxin XI (Fig. 3*D*), which is reflected in a backbone RMSD of 1.3 Å (residues Thr^65^–Glu^94^ of Dkk4 and residues Glu^1^–Lys^22^ and Leu^25^–Gly^32^ of Jingzhaotoxin XI, PDB code 2A2V). In addition, the locations of all three disulfides in this subdomain of CRD1 are fully conserved in the ICKs.

### Characterization of the interactions of Dkk proteins with LRP6 E1E2

A number of groups have previously characterized the binding of full-length Dkk1 to LRP6 E3E4, resulting in the identification of a single, high-affinity site (*K_D_* of 21–67 nm) (5, 9–11). It has also been reported that full-length Dkk1 binds with high affinity to a single site on LRP6 E1E2 (*K_D_* of 22–64 nm) (5, 11). Similarly, a study showed that full-length Dkk2 binds tightly to single sites on both LRP6 E1E2 and E3E4, with comparable *K_D_* values of 53 and 38 nm, respectively (11). In addition, several groups have shown that the isolated CRD2 region of Dkk1 binds with similar high affinity to the interaction site on LRP6 E3E4 (*K_D_* of 50–71 nm), identifying this domain as the principal interaction site with the E3E4 region of LRP6 (9, 10). In contrast, there is considerable uncertainty regarding the region of Dkk1 responsible for the high-affinity interaction with LRP6 E1E2, with conflicting reports of tight binding to the isolated CRD1 and CRD2 domains (9, 10).

Importantly, it has been shown that short peptides corresponding to a region close to the N terminus of Dkk1 and containing a conserved N*X*I(R/K) motif bind with medium affinity to LRP6 E1 (*K_D_* ∼6 μm). This study also reported considerably higher affinity interactions for both full-length Dkk1 and Dkk2 binding to LRP6 E1 (*K_D_* values of 27 and 53 nm, respectively), which identifies LRP6 E1 as the high-affinity Dkk binding site within the E1E2 region of LRP6 and indicates that CRD1 and/or CRD2 is required for a tight interaction (11).

**Figure 4.**
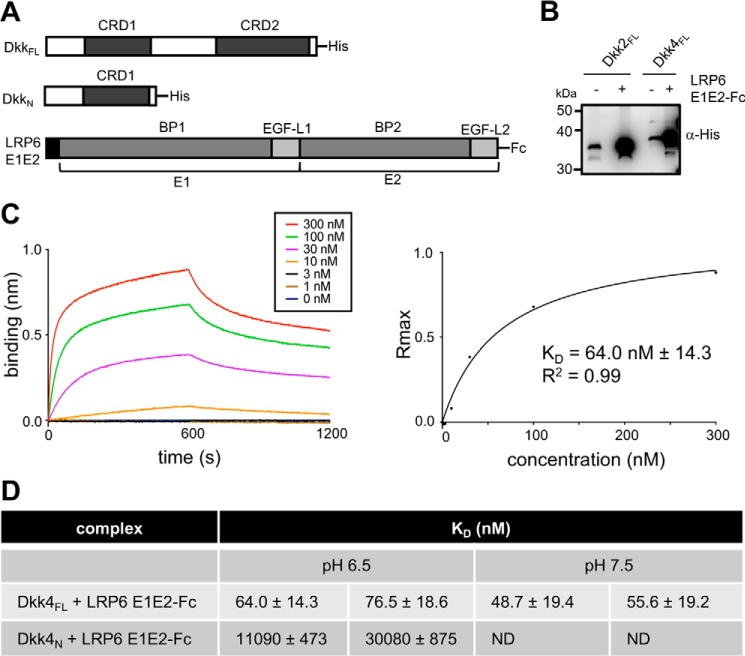
**Dkk4 binds with high affinity to LRP6 E1E2.** *A*, *schematic representations* of the human His-tagged Dkk4_FL_ and Dkk4_N_ and LRP6 E1E2-Fc expression constructs. *B*, Western blot analysis of pulldown assays, illustrating the binding of His-tagged Dkk proteins to LRP6 E1E2-Fc captured on protein A beads. *C*, representative biolayer interferometry sensorgrams for His-tagged Dkk4_FL_ binding to immobilized LRP6 E1E2-Fc. Association and dissociation phases of sensorgrams are shown for a range of indicated concentrations of His-tagged Dkk4_FL_, together with the steady-state binding curve derived from the maximum response (*R*_max_) observed. The *K_D_* reported was obtained by fitting to a one-site binding model using Prism version 6.0. *D*, a summary of biolayer interferometry *K_D_* measurements from two individual experiments and representative of a minimum of three or more independent measurements. Errors shown are the S.E. calculated for individual fitted curves using Prism version 6.0. *ND*, no data obtained.

To further understanding of which regions of Dkk proteins bind to LRP6 E1E2 and to extend previous characterization to include Dkk4, we carried out a series of experiments to determine the region of Dkk proteins responsible for high-affinity binding to LRP6 E1. Initially, pulldown assays were used to confirm the ability of Dkk4_FL_ and Dkk2_FL_ to form a tight complex with LRP6 E1E2-Fc (Fig. 4*B*). Subsequent biolayer interferometry (BLI) studies revealed that Dkk4_FL_ bound to LRP6 E1E2-Fc with a *K_D_* of 64–77 nm at pH 6.5 (Fig. 4, *C* and *D*), which is comparable with values reported previously for Dkk1 and Dkk2 binding to LRP6 E1E2 (5, 11).

Comparable binding experiments at pH 7.5 revealed no significant effect on the affinity of Dkk4 binding to LRP6 E1E2 (Fig. 4*D*) (Fig. S4). As perhaps expected from previous reports, the isolated Dkk4_N_ region and LRP6 E1E2 showed a much weaker interaction (*K_D_* of 11–30 μm), which is in good agreement with the affinities reported for short peptides containing an N-terminal N*X*I(R/K) motif binding to LRP6 E1 or E1E2 (11, 31).

**Figure 5.**
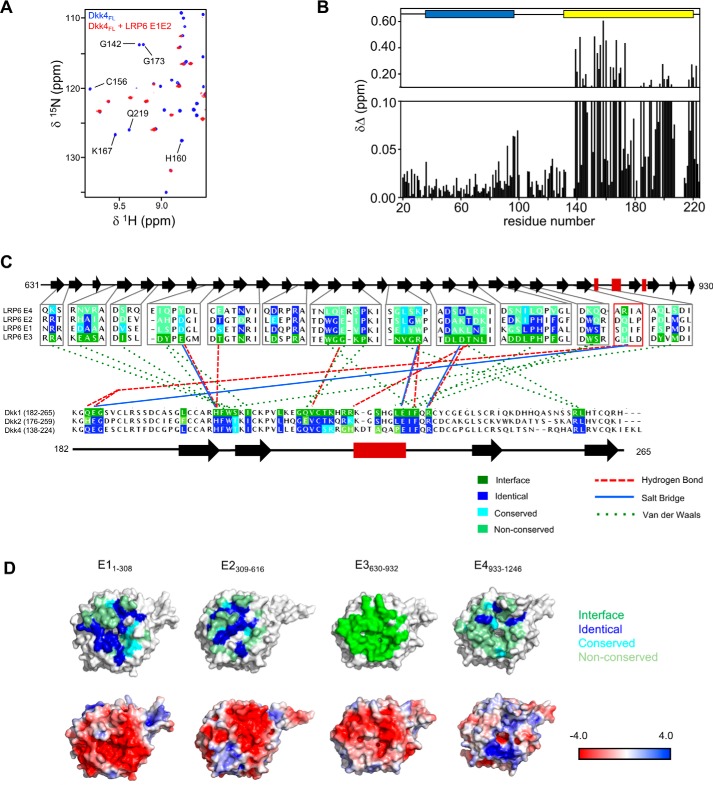
**Mapping of the LRP6 E1E2 interaction site on Dkk4.** *A*, overlay of selected regions from the ^15^N/^1^H TROSY spectra of uniformly ^15^N/^13^C/^2^H-labeled Dkk4_FL_ acquired in the absence (*blue*) and presence (*red*) of a 10% molar excess of unlabeled LRP6 E1E2. A selection of the well-resolved signals from Dkk4 CRD2 that are lost on binding to LRP6 E1E2 are labeled by residue type and number. *B*, minimum chemical shift perturbation observed for backbone amide groups of His-tagged Dkk4_FL_ induced by the addition of LRP6 E1E2. The positions of CRD1 and CRD2 are shown *above* the histogram as *blue* and *yellow boxes*, respectively. *C*, a summary of the key interactions observed between LRP6 E3 (*gray boxes*), a four-residue stretch of E4 (*red box*), and Dkk1 CRD2 in the highest-resolution reported crystal structure (PDB accession code 3S2K). Only the regions of LRP6 E1–E4 located at the binding interface are included in the multiple-sequence alignment shown. The multiple-sequence alignments shown for LRP6 E1–E4 and Dkk1–4 CRD2 indicate high conservation of the E3–Dkk1 CRD2 binding interfaces on LRP6 E1 and E2, and of the LRP6-binding site on the CRD2 domain of Dkk2 and Dkk4. A *schematic* of the regular secondary structure in both proteins is shown *above* or *below* the relevant multiple-sequence alignments, with *black arrows* and *red rectangles* representing β-sheets and α-helices, respectively. Residues found at the interaction site are highlighted in *green. D*, surface views of the potential Dkk CRD2-binding surface on LRP6 E1–E4. Electrostatic potential and sequence conservation compared with the Dkk1 CRD2 interaction site on LRP6 E3 are shown on the crystal structures of E1, E2, and E4 (PDB codes 4DG6 for LRP6 E1E2 and 3S2K for LRP6 E3E4).

To obtain a direct insight into the binding sites for LRP6 E1E2 on Dkk4, we carried out NMR chemical shift perturbation experiments using uniformly ^15^N/^13^C/^2^H-labeled Dkk4_FL_ and unlabeled LRP6 E1E2 at pH 6.5. ^15^N/^1^H TROSY experiments were acquired in the presence and absence of LRP6 E1E2 to monitor binding-induced changes in Dkk4_FL_ backbone NMR signals, which were used to determine the regions of Dkk4 involved in these interactions (Fig. 5*A*). The signals from Dkk4 CRD2 were not detectable in the LRP6 E1E2-bound Dkk4 spectra. The loss of these signals is indicative of the formation of a large tight complex (>100 kDa), resulting in substantial broadening of the NMR signals from the tightly associated region of Dkk4 (CRD2). Backbone chemical shift perturbations induced by LRP6 E1E2 binding showed that a small number of signals from residues in both the flexible N terminus and CRD1 were slightly affected by LRP6 E1E2 binding, indicative of a weak transient interaction consistent with the *K_D_* determined for the isolated N-terminal region of Dkk4 (Figs. 4*D* and 5*B*).

Our NMR data confirm a high-affinity interaction between LRP6 E1E2 and Dkk4 CRD2. The interaction between Dkk1 CRD2 and LRP6 E3 has been characterized in detail by X-ray crystallography. We therefore wished to examine the sequence conservation of the four LRP6 β-propeller regions to gain further insights into the ability of the CRD2 of Dkk proteins to interact with multiple propellers. Comparison of the structures and sequences of the individual LRP6 E1, E2, and E4 regions with E3 (PDB codes 4DG6 and 3S2K) shows that the CRD2-binding site appears to be relatively highly conserved on E1 and E2 (Fig. 5, *C* and *D*). The surface charge features are also fairly highly conserved on the faces of E1 and E2, equivalent to the Dkk1 CRD2 interaction site on E3 but not on E4 (Fig. 5*D*). Interestingly, E1 shows the closest structural homology to E3, whereas E2 has an extended loop that would partially obstruct the potential Dkk-binding site. Our analysis reveals that the Dkk CRD2 binding interface on E3 is largely conserved on E1, which is consistent with similar affinities determined for Dkk proteins binding to the E1E2 and E3E4 regions of LRP6.

Similar structural and sequence comparisons revealed that residues from the Dkk1 CRD2 domain involved in key interactions with LRP6 E3 and presumably E1 are very highly conserved in Dkk2 and Dkk4 (Fig. 5*C*). These residues are not conserved in Dkk3, which likely explains why Dkk3 is unable to bind LRP6 and inhibit Wnt signaling.

### Characterization of the interactions of Dkk proteins with Kremen extracellular domains

Previous studies have reported high-affinity binding of both full-length mature Dkk1 and Dkk2 to membrane-associated Krm1 and Krm2 (*K_D_* of 0.3–0.4 nm). For Krm1, there was also evidence of a slightly lower affinity binding site for both Dkk proteins, with a *K_D_* of 3 nm (12). Recently, immobilized Krm1 ECD was reported to bind to full-length mature Dkk1 and its isolated CRD2 at pH 7.5 with comparable affinity (*K_D_* values of 1.2 and 0.4 μm, respectively), but significantly more weakly (>10^3^-fold) than reported for membrane-bound Krm1. No detectable interaction was seen between the Dkk1 CRD1 and Krm1 ECD (13).

**Figure 6.**
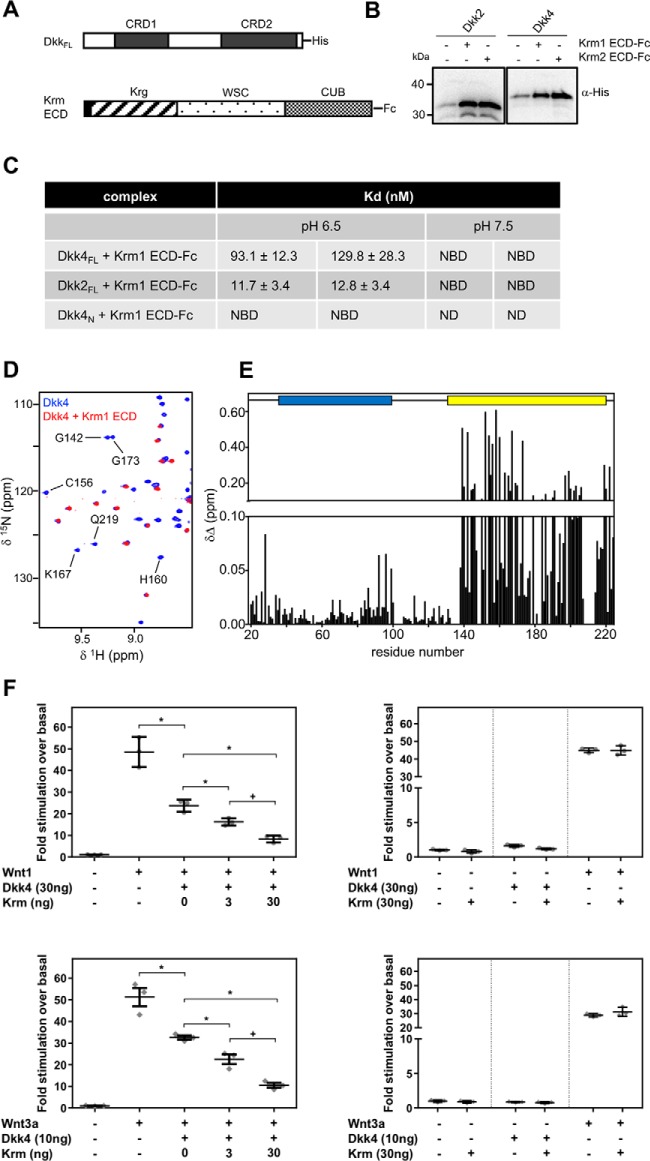
**Dkk2 and Dkk4 bind with high affinity to Krm ECD.** *A*, *schematic representations* of human His-tagged Dkk_FL_ and Krm ECD-Fc constructs. *B*, Western blot analysis of pulldown assays illustrating the binding of His-tagged Dkk_FL_ proteins to Krm1 ECD-Fc and Krm2 ECD-Fc captured on protein A beads. *C*, a summary of biolayer interferometry *K_D_* measurements from two individual experiments and representative of a minimum of three or more independent experiments. *Error bars*, S.E. calculated for individual fitted curves using Prism version 6.0. *NBD*, no binding detected; *ND*, no data obtained. *D*, overlay of a selected region from the ^15^N/^1^H TROSY spectra of uniformly His-tagged ^15^N/^13^C/^2^H-labeled Dkk4_FL_ acquired in the absence (*blue*) and presence (*red*) of a 10% molar excess of unlabeled Krm1 ECD. A selection of the well-resolved signals from Dkk4 CRD2 that are lost on binding to the Krm1 ECD are *labeled* by residue type and number. *E*, minimum chemical shift perturbation observed for backbone amide groups of His-tagged Dkk4_FL_ induced by the addition of Krm1 ECD. The positions of CRD1 and CRD2 are shown *above* the histogram as *blue* and *yellow boxes*, respectively. *F*, scatter plots of Wnt reporter assays illustrating synergistic inhibition of Wnt-dependent signaling by Dkk4 and Krm1. HEK293 Tcf-Luc cells were transiently co-transfected with plasmids to express Wnt1 (*top left*) or Wnt3a (*bottom left*), together with an amount of plasmid encoding Dkk4 determined to give partial inhibition of Wnt-dependent signaling and increasing amounts of a Krm1 expression plasmid. Appropriate control data are also shown for both the Wnt1 and Wnt3a experiments (*top* and *bottom right*, respectively). The results are presented as scatter plots showing individual data points, with *bars* indicating mean ± S.D. (*error bars*). Analysis of variance pairwise comparison tests confirm significant differences (*p* < 0.05) between Dkk4 alone and Dkk4 with both levels of Krm1 (*) and in the responses with different Krm doses (+).

As with the detailed characterization of Dkks binding to LRP6 E1E2 reported here, a complementary combination of approaches was used to study the interaction between Dkk and Krm proteins (Fig. 6*A*). Pulldown assays clearly showed that both Dkk2_FL_ and Dkk4_FL_ form a tight complex with Krm1/2 ECD-Fc (Fig. 6*B*). Follow-on BLI studies revealed that Dkk4_FL_ bound to a Krm1 ECD-Fc fusion protein at pH 6.5 with a *K_D_* of 93–130 nm, whereas Dkk2 bound with slightly stronger affinity (*K_D_* ∼12 nm) under the same conditions (Fig. 6*C* and Fig. S5). Comparable investigations with the isolated Dkk4_N_ region showed no evidence of binding to Krm1 ECD-Fc (Fig. 6*C*). In contrast to the minimal effect of pH on Dkk4 binding to LRP6 E1E2, there was a substantial change in binding to the Krm1 ECD at pH 7.5, with both Dkk2_FL_ and Dkk4_FL_ showing no significant binding at concentrations of up to 1 μm.

The isolated Krm1 ECD was also prepared for NMR chemical shift perturbation experiments, and following removal of the Fc tag, we noted that the Krm1 ECD eluted in size-exclusion chromatography as two separate peaks, consistent with the molecular masses expected for a monomer (35 kDa) and dimer (70 kDa) (Fig. S5*C*). SDS-PAGE analysis under reducing and nonreducing conditions revealed little difference between the monomeric and dimeric Krm1 ECD fractions, indicating the formation of a noncovalent dimer (Fig. S5*D*). Dimerization of the Krm1 ECD might be mediated through the CUB domain, as there are many examples of proteins that dimerize through this domain (32), including complement proteases in which the CUB domain mediates dimerization and binding to target proteins. The recently solved crystal structure of the Krm1 ECD revealed a potential dimer interface formed by the CUB domain; however, it was unclear whether this was a consequence of crystal packing (13). It is interesting to note that in this study, over 80% of the Krm1 ECD produced for the crystallographic work was found to exist as aggregates, and no monomeric Krm2 ECD could be obtained (13). These reported observations, together with our data, strongly suggest that both the Krm1 and Krm2 ECD regions readily form multimeric species, implying that a dimer or higher order complex is likely to be the functional form present on cell surfaces. This has important implications for understanding the molecular mechanism of synergistic regulation of Wnt signaling by Krm and Dkk proteins, which are discussed later.

^15^N/^1^H TROSY experiments were acquired in the presence and absence of a molar excess of Krm1 ECD to identify changes in Dkk4_FL_ backbone NMR signals induced by complex formation, which were used to determine the regions of Dkk4 involved in binding to the Krm1 ECD (Fig. 6, *D* and *E*). As previously seen upon binding to LRP6 E1E2, signals from the Dkk4 CRD2 were not detectable after formation of a Dkk4–Krm1 ECD complex. The loss of these backbone amide signals is indicative of the formation of a large tight complex, which in the case of a 2:2 Dkk4/Krm1 ECD dimer would be in excess of 120 kDa, resulting in dramatic broadening of the NMR signals from the regions of Dkk4 tightly bound to the Krm1 ECD, rendering them undetectable. Interestingly, some small shifts were also observed within the flexible N-terminal region and CRD1 of Dkk4, perhaps suggesting a transient interaction of these regions with either the Krm1 ECD or Krm1-bound CRD2 of Dkk4.

Overall, our combined pulldown, biosensor, and NMR studies of the Krm1 and Krm2 ECD binding to full-length mature Dkk2 and Dkk4 are consistent with high-affinity interactions at pH 6.5 (*K_D_* values ∼12 and 111 nm, respectively). Interestingly, we observed no detectable binding at pH 7.5, which may point to a more important role for Krm modulation of Wnt signaling under specific circumstances associated with reduced local pH. In agreement with previous work, we observed no tight interaction of the Krm1 ECD with the isolated CRD1-containing N-terminal region of Dkk4; however, our NMR mapping experiments revealed a high-affinity interaction with Dkk4 CRD2, confirming the importance of this Dkk protein domain in mediating the interaction with Krm.

### Synergistic regulation of Wnt signaling by Dkk and Krm proteins

To assess the reported potential for synergistic regulation of Wnt signaling by Dkk and Krm proteins, we investigated the ability of Dkk1 and Dkk4 to inhibit Wnt signaling in the presence of Krm1. The data presented in Fig. 6*F* clearly demonstrate the ability of Krm1 to concentration-dependently enhance inhibition of Wnt1 or Wnt3a signaling by a submaximal amount of Dkk4. Comparable effects were also observed in experiments with Dkk1 and Krm1 (Fig. S6). The submaximal Dkk1 and Dkk4 amounts used for these experiments were chosen based on the titrations of Dkk1 and Dkk4, which are shown in Fig. S2. Our results therefore suggest a common synergy between Dkk and Krm proteins in the inhibition of Wnt signaling. These findings are consistent with an earlier study, which reported that Krm proteins could act synergistically with Dkk1 and -2 to regulate Wnt signaling (12). This functional cooperativity is believed to reflect the formation of a ternary complex of Krm, Dkk, and LRP6 (12).

**Figure 7.**
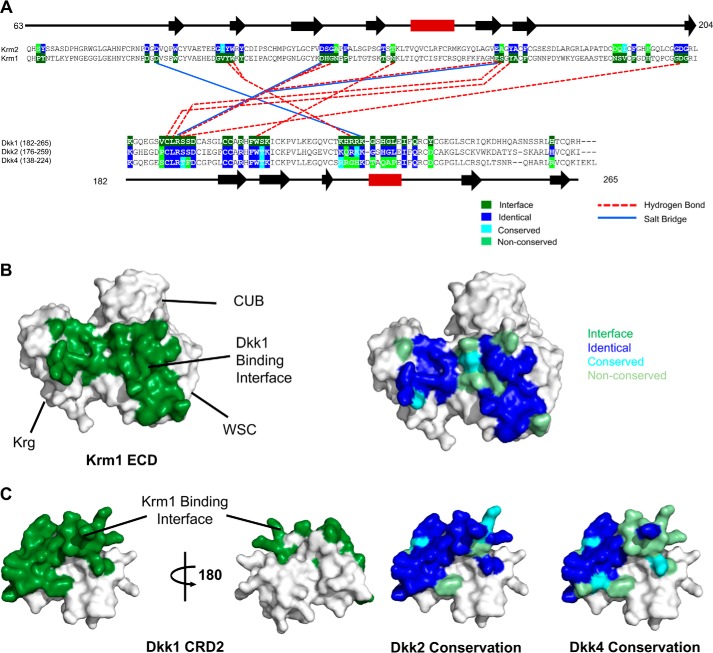
**Analysis of the reported Krm1 ECD and Dkk1 CRD2 interaction sites.** *A*, summary of the key hydrogen bond and salt bridge interactions implied by the Krm1 ECD and Dkk1 CRD2 interface observed in the low resolution crystal structure of the ternary complex (PDB code 5FWW). The complex features an extensive contact surface between the two proteins (1025 and 934 Å^2^ on Dkk1 CRD2 and Krm1 ECD, respectively), involving many van der Waals interactions. The multiple-sequence alignments shown for Krm1 and Krm2 ECD (*top*) and Dkk1, Dkk2, and Dkk4 CRD2 (*bottom*) indicate the high conservation of the binding interface between the two families of proteins. A *schematic* of the regular secondary structure, as seen in the crystal structure, is displayed *above* and *below* the sequence alignments with *black arrows* and *red rectangles* representing β-sheets and α-helices, respectively. *B*, *surface views* of the Krm1 ECD crystal structure (PDB code 5FWW) showing the location of the Dkk1 CRD2-binding site (*left panel*, highlighted in *green*) and conservation of the Krm1 residues in Krm2 at the Dkk1 CRD2-binding interface (*right panel*). *C*, surface views of the crystal structure of Dkk1 CRD2 (PDB code 5FWW) showing the location of the Krm1 ECD binding site (*left panels*, highlighted in *green*) and the sequence conservation of Dkk2 and Dkk4 residues at the Krm1 ECD binding interface (*right panel*).

To further understand the potential for different members of the Krm and Dkk families to form functional ternary complexes, residues located at the Dkk1-binding site on Krm1 (PDB code 5FWW) were compared with the equivalent residues of Krm2 (Fig. 7, *A* and *B*) (13). This revealed a high degree of sequence conservation between the two binding faces, with fewer than 7 residues at the interface being nonconserved. Similarly, analysis of the conservation of the Dkk1 CRD2 residues involved in binding to the Krm1 ECD (Fig. 7*C*) revealed that Dkk2 has the highest number of equivalent residues (82% identity), whereas these positions are poorly conserved in Dkk3. Interestingly, the equivalent Krm-binding residues in Dkk4 are only partially conserved with Dkk1; however, we still observed tight binding between the Krm1 ECD and Dkk4 in both BLI and NMR experiments (Figs. 6 and 7, *A* and *C*). Overall, the ability of both Krm proteins to form functional complexes with Dkk1, Dkk2, and Dkk4 appears to have been conserved, providing a diverse spectrum of potential Wnt-inhibitory complexes, perhaps reflecting a need for a spectrum of inhibitory potency and/or tissue specificity.

## Discussion

The Dkk family proteins act as important regulators of Wnt signaling. Although the nature of the interactions that Dkk1 makes with its partners Krm1 and the E3E4 region of LRP5/6 have been thoroughly characterized, surprisingly little is known of the molecular interactions underpinning Dkk2- and Dkk4-based inhibition of Wnt signaling. Similarly, the structure and interactions of the isolated CRD2 domain from Dkk1 and Dkk2 have been well characterized; however, no equivalent information has been reported for the CRD1 domain of any Dkk protein.

Here we report the first detailed structural and dynamics characterization of a full-length mature Dkk protein (Dkk4) and reveal that Dkk proteins consist of two completely independent, structurally homologous cysteine-rich domains (CRD1 and CRD2), joined by a highly flexible linker and containing a similarly dynamic N-terminal region. The structural homology identified between the two CRDs of the Dkk proteins was unexpected and had not been predicted by sequence comparisons. There is also a striking structural similarity between the N- and C-terminal subdomains of both CRD1 and CDR2 and between these Dkk subdomains and ICK proteins, which suggests that the cysteine-rich domains of Dkks and colipases might have evolved from a common ancient ancestor that contained just one subdomain. Duplication of the gene encoding this ancestral domain would have produced the original cysteine-rich domain equivalent to the examples now found in Dkk proteins and colipases. It seems likely that a second gene duplication event occurred in the Dkk ancestor, resulting in the emergence of a protein containing two structurally homologous cysteine-rich domains. Subsequent sequence and structural divergence of the two Dkk CRDs has allowed the evolution of distinct functional roles for CRD1 and CRD2.

The work reported here reiterates a key role for the CRD2 region of Dkks in mediating high-affinity interactions with Krm and LRP6 family proteins to regulate Wnt signaling. We discovered two potential novel binding sites in highly charged regions of the Dkk4 CRD1 surface, and it will be important for the field to uncover the precise roles and functional partners for CRD1 in the context of Wnt regulation.

A number of groups have reported studies of the interactions of Dkk1 with LRP6; however, a somewhat confused picture has emerged concerning the regions of Dkk1 involved in binding to the E1E2 region of LRP6. Our combined approach using biosensor and NMR investigations of both full-length mature Dkk4 and the isolated N-terminal CRD1-containing region underscores the importance of the C-terminal CRD2 region as the domain responsible for tight binding to LRP6 E1E2. We also observed a significantly weaker (∼170-fold), medium-affinity interaction mediated by the N-terminal region of Dkk4 alone. We believe this reflects binding through the nonstructured N*X*I(R/K) motif found close to the N terminus of Dkk4, which is analogous to the region of Dkk1 previously shown to bind with comparable affinity to LRP6 E1 (11). Analysis of the conservation of the Dkk1 CRD2-binding site identified on LRP6 E3 on E1, E2, and E4 highlights strong conservation of this site on E1, which also corresponds to the peptide N*X*I(R/K)-binding site found on E1. This suggests that the N-terminal N*X*I(R/K) motif and CRD2 domain of Dkk4 bind to the same site on LRP6 E1; however, the substantially higher affinity seen for the interaction with CRD2 would be expected to favor binding through this domain for the intact protein. This is reflected in relatively small perturbation of the backbone NMR signals from the N*X*I(R/K) region on binding of full-length mature Dkk4 to LRP6 E1E2 (Fig. 5*B*). Furthermore, the high sequence conservation of the CRD2 region in Dkk1, -2, and -4 strongly suggests that CRD2 will mediate a high-affinity interaction with LRP6 E1 for all three proteins.

Characterization of the binding of Dkk4 to the Krm1 ECD revealed a high-affinity interaction at pH 6.5 (Fig. 6 and Fig. S5), but surprisingly no interaction could be detected at pH 7.5. We also obtained similar findings with Dkk2, suggesting the potential for pH-dependent modulation of the functional synergy between Dkk and Krm proteins. Our NMR studies identify the CRD2 domain of Dkk4 as the primary interaction site for the Krm1 ECD and are consistent with a Krm1 ECD homodimer binding to two molecules of Dkk4 (120 kDa). The recently reported structure of the LRP6 E3E4–Dkk1 CRD2–Krm1 ECD ternary complex (13) shows the Dkk1 CRD2 contacting both the Krg and WSC domains of Krm1, with the region involved highly conserved on Krm2. Previous work has pointed to a strong synergy between either Dkk1 or Dkk2 and Krm1/2 proteins in the inhibition of Wnts that signal through interaction with both LRP6 E1 (Wnt1) and E3 (Wnt3a). We have similarly shown that both Dkk1 and Dkk4 synergize with Krm1 to inhibit Wnts that act via E1 and E3 of LRP6, suggesting a common mechanism of action across the Dkk and Krm proteins.

**Figure 8.**
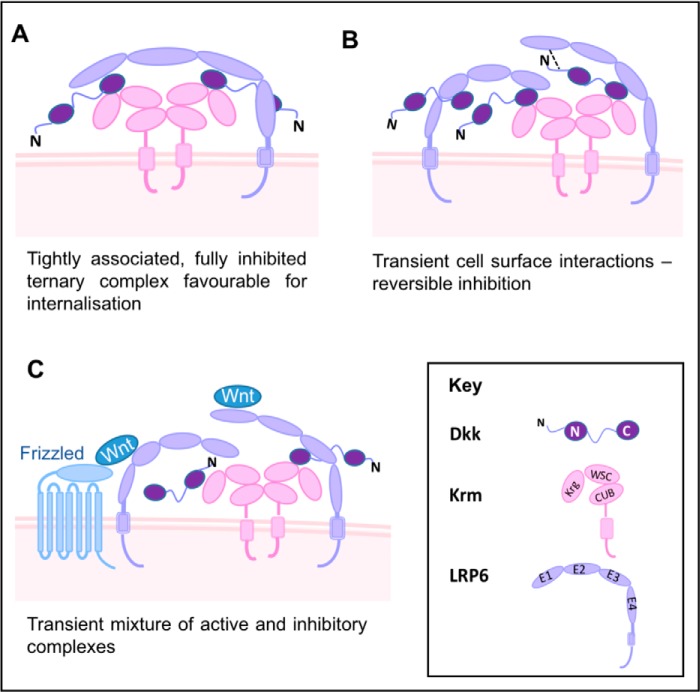
**Synergistic regulation of Wnt signaling by Dkk and Krm family proteins: Diversity of potential functional complexes formed.** *A–C*, possible schematic models for the interaction of Dkk, Krm, and LRP5/6 proteins on the cell surface, illustrating the potential for a wide spectrum of functional complexes to form to provide exquisite fine-tuning of Wnt-signaling activity. The diversity of potential complexes includes both high affinity Dkk–LRP5/6 inhibitory interactions mediated by the CRD2 domain of Dkk proteins and relatively low-affinity interactions involving the conserved N*X*I(R/K) motif located in the flexible N terminus of Dkks. The potential complexes shown highlight the tendency of the Krm ECD to form stable dimers.

The work described here and reported previously points to a complex picture of potential high- and low-affinity interactions among the Dkk, Krm, and LRP5/6 functional partners, strongly implying the formation of a range of functional complexes at the cell surface. To assist in developing an understanding of the synergistic regulation of Wnt signaling by Dkk and Krm proteins, we propose a model in which the CRD2 region of Dkks mediates their tight association with membrane-anchored dimers of the Krm ECD (Fig. 8). This would immobilize a pool of Dkk proteins on the cell surface, providing a relatively high local concentration and poised to interact with LRP5/6 to form Wnt signaling–inhibitory complexes, analogous to the formation of Wnt/LRP5/6 signalosomes that have been shown to initiate Wnt signaling (33). There are a diverse range of possibilities for inhibitory interactions with LRP5/6, which could facilitate delicate spatio-temporal control of this central signaling pathway. The portfolio of inhibitory complexes actually formed would be dependent upon the proteins present and therefore the cellular context.

When we combine our findings with those presented by Zebisch *et al.* (13), it becomes possible to refine the model of canonical Wnt inhibition by Dkk family proteins. It is now clear that there are multiple Dkk-binding sites on LRP5/6, several distinct LRP5/6 interaction sites on Dkk proteins, and at least three Dkks that bind to LRP5/6 (Fig. 8). The diversity of possible Dkk–LRP5/6 interactions indicated in the range of regulatory Krm–Dkk–LRP5/6 complexes proposed in Fig. 8 is therefore likely to be applicable to Dkk1, -2, and -4. The schematic model outlined here provides a mechanistic basis for the synergistic regulation of Wnt signaling by Dkk/Krm proteins, which provides the potential for exquisite fine-tuning of the inhibition of Wnt signaling.

As illustrated in Fig. 8*A*, one possibility is for a single molecule of LRP5/6 to interact with two Dkk proteins bound to a cell surface immobilized Krm dimer, with binding to the E1 and E3 regions of LRP5/6 mediated by the two Dkk CRD2 regions. Given the high affinity of the interactions with both E1 and E3, this complex would dissociate very slowly and would be suited to a functional situation where complete inhibition of Wnt signaling is required for an extended period of time. A number of studies have reported that ternary Krm–Dkk–LRP5/6 complexes are internalized through a Krm-dependent process (34, 35), which would be facilitated by the formation of tightly associated ternary complexes on the cell surface, as proposed here.

Another possible binding mode involves two Dkks bound to a single Krm dimer interacting with distinct LRP5/6 receptors. In this situation, either E1 or E3 on the receptor could mediate ternary complex formation, leaving the other free to interact with either a non-membrane-associated inhibitor or even Wnt proteins (Fig. 8, *B* and *C*). This type of ternary complex would be more transient and retain the potential to mediate Wnt signaling. The diversity of ternary Krm–Dkk–LRP5/6 complexes expected to form at the cell surface, with a wide spectrum of stability and degree of inhibition, provides a mechanism for very fine-tuning of the regulation of Wnt signaling. This potential for exquisite fine-tuning of activity may be key to the central role of Wnt signaling in a diverse range of processes, where a simple on/off system would not be sufficient. Further detailed molecular characterization of the nature and dynamics of these regulatory complexes is likely to reveal new opportunities for therapeutic intervention and provide greater understanding of tissue-specific Wnt-dependent processes.

In summary, we present the first detailed structural and dynamics study of a full-length mature Dkk protein with the N-terminal signal sequence absent. Despite the divergence in primary amino acid sequence, we discovered that the CRD1 and CRD2 domains of Dkk4 share great structural similarity. The identification of two putative binding sites on the CRD1 domain highlights the potential existence of novel interacting partners for the CRD1 domain of Dkk family proteins. Our comparison of the binding of full-length mature Dkk4 and Dkk4_N_ to LRP6 E1E2 and Krm1 ECD has led to a deeper understanding of the mechanism of interaction of Dkk family proteins with their cognate receptors and underscores the importance of the CRD2 motif in mediating high-affinity binding. Detailed structural and biophysical data for the different Krm–Dkk–LRP6 complexes are key to understanding the molecular basis of their inhibition of Wnt signaling, and our insights could inform the development of new therapeutics.

## Experimental procedures

### Protein expression and purification

Details of the expression and purification of the Dkk, LRP6, and Krm proteins studied are provided in the supporting material.

### CD spectroscopy

CD was used to assess the secondary structure and thermal stability of proteins. Samples of Dkk2_FL_ and Dkk4_FL_ (10 μm) in 25 mm bis-Tris, 100 mm NaCl, pH 6.5, were prepared and analyzed using a 0.5-mm quartz cuvette. The instrument used was a Chirascan Plus spectrophotometer (Applied Photophysics), and the Pro-Data Chirascan software (Applied Photophysics) was used to collect and perform basic analysis. Data were collected in the far-UV region from 195 to 260 nm at 1-nm and 0.5-s intervals. Before analysis, the CD spectra were corrected for buffer absorbance, and the raw data were converted to mean residue molar ellipticity ([θ]_MRME_).

For temperature denaturation of samples, a temperature probe was inserted into the sample, and temperature ramping was enabled from 15 to 95 °C with 0.5 °C increments and a 45-s settling time at each point. Four repeats were also recorded at each point. Upon completion of the temperature melt, the signal was converted to [θ]_MRME_, and the data were exported to Prism version 6.0h. The relationship between the CD signal and temperature was plotted at the wavelength where the most intense changes were observed and fitted to a sigmoidal curve.

### Analytical size-exclusion chromatography (SEC)

Analytical SEC for apparent molecular weight determination of monomeric and dimeric Krm1 ECD proteins was performed on a Superdex^TM^ 75 10/300 GL column (GE Healthcare) connected to an ÄKTA FPLC system (GE Healthcare), which was fitted with a 1-ml sample loop. The column was pre-equilibrated with Krm1 ECD SEC buffer (50 mm Na_2_HPO_4_, 100 mm NaCl, pH 7.5). Samples of 3 μm Krm1 ECD were made up in Krm1 ECD SEC buffer before injecting samples (500 μl) onto the column, which was run for 2 column volumes with a flow rate of 0.6 ml/min while monitoring the *A*_280_. A calibration was performed for the SEC column to allow the estimation of molecular weights. The calibration was done using the Gel Filtration LMW Calibration Kit (GE Healthcare). Globular protein standards were made up in 50 mm Na_2_HPO_4_, 100 mm NaCl, pH 7.5, at the manufacturer's recommended concentrations. The relationship between the logarithm of the molecular weight and elution volume for the globular standard was used to plot a standard curve. The resulting line of best fit was used to calculate apparent molecular weights of the Krm1 ECD species.

### LRP6 FACS-binding assay

FACS-binding assays were performed essentially as described previously (31). Cells were seeded into poly-d-lysine–coated 6-well plates (1.2 × 10^6^ cells/well) and allowed to attach before being transiently transfected with 4 μg/well of empty vector (mock) or vector encoding full-length LRP6, using Lipofectamine 2000 (Invitrogen^TM^) according to the manufacturer's instructions. Cells were harvested nonenzymatically typically on the day after transfection. For detection of Dkk binding to cell surface LRP6, >2 × 10^5^ cells were labeled with His_6_-tagged Dkk1 (R&D Systems), Dkk2 (R&D Systems), refolded Dkk2_FL_, and refolded Dkk4_FL_ (between 11 and 110 nm). A negative control with no Dkk was also set up. The cells were incubated with the Dkk proteins for 2 h at 4 °C in PBS buffer containing 10% (v/v) fetal calf serum, 1% (v/v) BSA, and 0.1% (v/v) NaN_3_. Cells were washed and stained with an anti-His-PE (phycoerythrin) antibody (Miltenyi Biotech) for 45 min at 4 °C. Cells were washed, and PE fluorescence was analyzed via excitation at 488 nm and emission at 585 nm using a FACSCalibur (BD Biosciences).

### Wnt reporter assays

HEK Tcf-Luc cells were seeded at a density of 5 × 10^4^ cells/well in 96-well plates in 70 μl of Dulbecco's modified Eagle's medium containing 0.5% fetal calf serum, 2 mm l-Glu, and 1× nonessential amino acids. After a 3-h incubation at 37 °C, cells were treated with recombinant protein or were transiently transfected using 200 ng of plasmid DNA/well complexed with Lipofectamine 2000 in Opti-MEM. Following a 24–48-h incubation at 37 °C, Wnt signaling activity was assessed by adding Steady-Glo Reagent (Promega) to the wells in equal volume to the medium. After a 20-min incubation at room temperature, the emission of light catalyzed by luciferase was measured using a Synergy 2 plate reader (BioTek Instruments, Inc.).

### NMR spectroscopy

NMR spectra were acquired from 0.35-ml samples of 150 μm ^15^N/^13^C/^2^H-labeled Dkk4_FL_ and 90–230 μm ^15^N- or ^15^N/^13^C-labeled Dkk4_N_. All samples were in a 25 mm Na_2_HPO_4_, 100 mm NaCl, 0.02% (w/v) NaN_3_ buffer at pH 6.5, containing either 5% D_2_O, 95% H_2_O or 100% D_2_O as appropriate. All NMR data were acquired at 35 °C on either a 600-MHz Bruker AVIII, 800-MHz Bruker AVII, or 950-MHz Bruker Avance III HD spectrometer fitted with cryogenically cooled probes.

The 2D and 3D spectra recorded to obtain sequence-specific backbone assignments for Dkk4_FL_ were as follows: ^15^N/^1^H TROSY and ^15^N/^13^C/^1^H TROSY-based HNCACB, HN(CO)CACB, HNCA, and HNCO (reviewed in Ref. 36). Typical acquisition times in F_1_ and F_2_ for the 3D experiments were 22 ms for ^15^N and 9.5 or 30 ms for ^13^C, with an acquisition time of 70 ms in F_3_ (^1^H). The majority of the 3D spectra were collected over ∼60 h. The HNCACB and HN(CO)CACB spectra were collected for ∼60 h using nonuniform sampling with the data sets sparsed at 27%. Typical acquisition times for the ^15^N/^1^H TROSY experiments were 50 ms in F_1_ (^15^N) and 80 ms in F_2_ (^1^H), with the spectra acquired for 1.5 h.

The 2D and 3D spectra recorded to obtain sequence-specific assignments and structural constraints for Dkk4_N_ were as follows: ^1^H-^1^H TOCSY with a mixing time of 60 ms, ^1^H-^1^H NOESY with an NOE mixing time of 150 ms; ^15^N/^1^H TROSY, NOESY-HSQC with an NOE mixing time of 250 ms; ^13^C/^1^H HSQC, HCCH-TOCSY with a mixing time of 13 ms, HSQC-NOESY with NOE mixing times of 150 and 250 ms; and ^15^N/^13^C/^1^H TROSY-based HNCACB, HN(CO)CACB, and HNCO, and a standard HBHA(CO)NH (reviewed in Ref. 36). Typical acquisition times in F_1_ and F_2_ for the 3D experiments were 14–22 ms for ^15^N, 7–30 ms for ^13^C, and 13–16 ms for ^1^H, with an acquisition time of 60–80 ms in F_3_ (^1^H). The majority of the 3D spectra were collected over ∼84 h, 2D ^1^H-^1^H experiments over 36 h, and ^15^N/^1^H and ^13^C/^1^H HSQC spectra over about 1 h. The HN(CO)CACB, HBHA(CO)NH, HCCH-TOCSY, and HSQC-NOESY (250-ms mixing time) experiments were collected over ∼66 h using nonuniform sampling with the data set sparsed at 25–50%. Typical acquisition times in 2D experiments were either 60 ms (^15^N), 9 ms (^13^C), or 60 ms (^1^H) in F_1_ and 80 ms in F_2_ (^1^H).

The NMR data were processed using either NMRPipe (37) or Topspin version 3.2 (Bruker Biospin Ltd.) with linear prediction used to extend the effective acquisition times by up to 1.5-fold in F_1_ and F_2_. The nonuniform sampled data were reconstructed using either MddNMR or NESTA-NMR (38, 39). All spectra were analyzed using the NMRFAM-Sparky version 3.115 package (40).

### Structural calculations

The family of converged Dkk4_N_ structures was determined in a two-stage process using the program CYANA version 2.1 (41, 42). Initially, the combined automated NOE assignment and structure determination protocol (CANDID) was used to automatically assign the NOE cross-peaks identified in 2D NOESY and 3D ^15^N- and ^13^C-edited NOESY spectra and to produce preliminary structures. This approach provides a completely unbiased assignment of the NOE peaks. Subsequently, several cycles of simulated annealing combined with REDAC were used to produce the final converged Dkk4_N_ structures. In addition to the NOE data, disulfide bond constraints for the five mapped disulfides and backbone torsion angle constraints derived from the prediction program TALOS-N were included in both stages of the calculation (20). Two manually assigned unambiguous NOEs (between Met^78^ Qϵ and Phe^52^ Qϵ or Hζ) were included in the final rounds of the CANDID calculation. Hydrogen bond constraints, involving 6 residues with slowly exchanging backbone amide signals (residues Leu^84^, Val^86^, Val^89^, Thr^91^, Cys^73^, and Cys^90^) were progressively added to the CANDID and REDAC calculations as the acceptor became apparent. Analysis of the final family of structures obtained was carried out using the programs CYANA version 2.1, MOLMOL, and PyMOL (Schroedinger LLC, New York) (41, 42).

### Secondary chemical shift calculations

Secondary chemical shifts were calculated for the ^1^H^N^ and ^15^N^H^ atoms for Dkk4_FL_ by subtracting experimentally obtained chemical shift values from predicted random coil chemical shift values. Absolute values were calculated for the combined ^1^H^N^-^15^N^H^ secondary chemical shift according to √((Δδ_HN_)^2^ + (Δδ_N_·α)^2^), where Δδ_HN_ and Δδ_N_ correspond to the differences in ^1^H^N^ and ^15^N^H^ chemical shifts between actual and predicted random coil values, and α_N_ is a scaling factor of 0.2 required to account for differences in the range of amide proton and nitrogen chemical shifts. Predicted random coil chemical shift values were calculated for Dkk4_FL_ at 308 K and pH 6.5 using the online software available at https://spin.niddk.nih.gov/bax/nmrserver/Poulsen_rc_CS/.^8^

### Heteronuclear NOE measurements

^15^N{1H}-NOE values for the backbone amide signals of Dkk4_FL_ were determined from a pair of interleaved spectra recorded with or without presaturation of the amide proton resonances during the relaxation delay. Spectra were acquired with a 360-μl sample of 110 μm Dkk4_FL_ in 25 mm Na_2_HPO_4_, 100 mm NaCl, pH 6.5, containing 5% (v/v) D_2_O. The spectra used to determine the ^15^N{1H}-NOE values for the Dkk4_FL_ backbone amide signals were recorded using an 800-MHz Bruker Avance II spectrometer with acquisition times of 80 and 90 ms in F_2_ (^1^H) and F_1_ (^15^N), respectively. Spectra were recorded with an NOE mixing time of 75 ms. Data were processed using NMRPipe (37) and analyzed using NMRFAM-Sparky version 3.115 (40). The values of the ^15^N{1H}-NOEs were determined from the peak heights measured in spectra recorded with (*I_s_*) or without (*I_o_*) presaturation of the ^1^H^N^ resonances according to (*I_s_* − *I_o_*)/*I_o_* (18, 19).

### NMR minimal shift mapping of binding sites

The minimal shift approach was used to determine the changes in the positions of Dkk4_FL_ NMR signals resulting from LRP6 E1E2 and Krm1 ECD binding (43, 44). The minimum change in the position for all backbone amide peaks between the free and receptor-bound Dkk4_FL_ was obtained by calculating the combined chemical shift difference in ^1^H^N^ and ^15^N^H^ for each assigned peak in the ^15^N/^1^H TROSY spectrum of the free protein compared with all peaks observed in the TROSY spectra of the complexes. The combined amide proton and nitrogen chemical shift difference (Δδ) was defined according to the calculation, Δδ = √((Δδ_HN_)^2^ + (Δδ_N_·α_N_)^2^), where Δδ_HN_ and Δδ_N_ correspond to the differences in ^1^H^N^ and ^15^N^H^ chemical shifts between pairs of compared TROSY peaks, and α_N_ is a scaling factor of 0.2 required to account for differences in the range of amide proton and nitrogen chemical shifts. For each individual TROSY peak, the minimal shift induced by ligand binding was taken as the lowest possible combined shift value (Δδ).

### Pulldown binding assays

Pulldown assays using protein A-agarose beads (Pierce^TM^) were conducted by capturing LRP6 E1E2-Fc or Krm ECD-Fc proteins before the addition of Dkk_FL_ proteins. For each assay, 400 μl of LRP6 E1E2-Fc (100 nm) in PBS with 0.5% (v/v) NP-40 and 3 μm BSA or 400 μl of cell culture medium containing expressed Krm ECD-Fc proteins was added to 1.5-ml Eppendorf® tubes. 20 μl of protein A beads were added to the tubes and incubated with tumbling for 1 h at 4 °C. Dkk2_FL_ and Dkk4_FL_ were then added at a final concentration of 200 nm. Tubes containing only Dkk proteins were set up as controls to monitor nonspecific binding to the beads. The tubes were further incubated with tumbling for 1 h at 4 °C. Subsequently, the beads were pelleted by centrifugation (21,000 × *g* for 20 s at room temperature). The supernatant was removed, and the beads were washed three times by resuspending in 1 ml of PBS, 0.5% (v/v) Nonidet P-40, 3 μm BSA and gently inverting for 30 s. After each wash, the beads were centrifuged (21,000 × *g* for 20 s at room temperature), the supernatant was removed, and fresh buffer was added.

Following the final wash step, the supernatant was removed, and 50 μl of reducing SDS-PAGE sample buffer was added, and the samples were analyzed by Western blotting. A primary anti-His antibody (Bethyl Laboratories, Inc.) and secondary peroxidase AffiniPure goat anti-rabbit IgG antibody (Jackson ImmunoResearch Laboratories, Inc.) were used to detect the His_6_ tag of Dkk_FL_ proteins and reveal binding to LRP6 E1E2 or Krm proteins.

### BLI binding assays

To obtain the equilibrium dissociation constants (*K_D_*) for Dkk2_FL_, Dkk4_FL_, and Dkk4_N_ binding to their functional partners, LRP6 E1E2 and Krm1 ECD, BLI experiments were carried out using AHC biosensors. Experiments were conducted in 25 mm Na_2_HPO_4_, 100 mm NaCl, 0.05% (v/v) Triton X-100, pH 6.5 or 7.5. Later experiments conducted with Dkk4_N_ also included 0.1% (w/v) BSA in the experiment buffer to decrease nonspecific binding.

Briefly, experiments were set up as follows. Biosensors were dipped into buffer for 120 s to acquire a baseline before the loading step, where the biosensors were dipped into a solution containing LRP6 E1E2-Fc (200 nm), Krm1 ECD-Fc (from supernatants), or a negative control of just buffer. For Krm1 ECD-Fc, a second baseline was recorded for 300 s with cell culture medium without the presence of Krm1 ECD-Fc before loading the Krm1 ECD-Fc. The loading step for the functional partners was carried out over 600 s or until a threshold of 1.5 nm had been reached. A second baseline with the experiment buffer was recorded for 120 s before measuring the association of Dkk proteins by dipping the biosensors into the wells containing Dkk2_FL_, Dkk4_FL_ (1000, 300, 100, 30, 10, 1, or 0 nm), or Dkk4_N_ (100, 30, 10, 3, 1, 0.3, 0.1, or 0 μm) for 600 s. Dissociation of the bound Dkk proteins was monitored by dipping the biosensors into wells containing buffer for a further 600 s.

The raw data points from the Octet® data analysis software (fortéBio) were exported into Prism (version 6.0h). The *K_D_* values were derived from steady-state equilibrium analysis by fitting the steady-state binding levels observed for a range of Dkk protein concentrations to a one-site binding model using Prism.

## Author contributions

S. P., A. M. B., F. W. M., A. C., A. J. H., M. K. R., L. C. W., G. H., and M. D. C. designed the study; S. P., A. M. B., D. G., S. L. S., S. B., F. W. M., P. W. A., P. S. R., P. M. S., C. D., A. C., R. J. T., C. E. P., A. J. H., M. K. R., L. C. W., G. H., and M. D. C. performed the experiments; S. P., A. M. B., F. W. M., C. E. P., A. J. H., M. K. R., L. C. W., G. H., and M. D. C. analyzed the data; and S. P., A. M. B., M. K. R., G. H., L. C. W., and M. D. C. wrote the paper.

## Supplementary Material

Supporting Information

## Footnotes

8 Please note that the JBC is not responsible for the long-term archiving and maintenance of this site or any other third party hosted site.

## Footnotes

7 The abbreviations used are:

Dkk: Dickkopf
CRD: cysteine-rich domain
Krm: Kremen
REDAC: redundant dihedral angle constraints
CANDID: combined automated NOE assignment and structure determination protocol
ECD: extracellular domain
Krg: kringle
2D and 3D: two- and three-dimensional, respectively
RMSD: root mean square deviation
PDB: Protein Data Bank
IGFBP-4: insulin-like growth factor-binding protein-4
ICK: inhibitor cysteine knot
BLI: biolayer interferometry
bis-Tris: 2-[bis(2-hydroxyethyl)amino]-2-(hydroxymethyl)propane-1,3-diol
SEC: size-exclusion chromatography.
