# Supplementary material for: Structural and functional analysis of Dickkopf 4 (Dkk4): New insights into Dkk evolution and regulation of Wnt signaling by Dkk and Kremen proteins
Source: J Biol Chem. 2018 Jun 20;293(31):12149–66. doi: 10.1074/jbc.RA118.002918 (PMC6078440; doi:10.1074/jbc.RA118.002918)
Supplement: Supporting Information [file supp_293_31_12149__index.html]

Structural and functional analysis of Dickkopf 4 (Dkk4): new insights into Dkk evolution and regulation of Wnt signalling by Dkk and Kremen proteins — Structure, Dynamics and Functional Complexes of Dkk4 — Structural and functional analysis of Dickkopf 4 (Dkk4): New insights into Dkk evolution and regulation of Wnt signaling by Dkk and Kremen proteins — Structure, dynamics, and functional complexes of Dkk4 — Supporting Information 

# Structural and functional analysis of Dickkopf 4 (Dkk4): New insights into Dkk evolution and regulation of Wnt signaling by Dkk and Kremen proteins

## Supporting Information

- Supporting Information - Supporting table, figures and methods
