# Supplementary material for: Structural and functional analysis of Dickkopf 4 (Dkk4): New insights into Dkk evolution and regulation of Wnt signaling by Dkk and Kremen proteins
Source: J Biol Chem. 2018 Jun 20;293(31):12149–66. doi: 10.1074/jbc.RA118.002918 (PMC6078440; doi:10.1074/jbc.RA118.002918)
Supplement: Supporting Information [file supp_RA118.002918_136683_1_supp_155545_pbkmjh.pdf]

Structural and functional analysis of Dickkopf 4 (Dkk4): new insights into Dkk evolution and regulation of Wnt signalling by Dkk and Kremen proteins.

Saleha Patel,[a,b,1] Alice M. Barkell,[a,b,1] Deepti Gupta,[c] Sarah L. Strong,[a,b] Shaun Bruton,[a,b] Frederick W. Muskett,[a,b] Philip W. Addis,[a,b] Philip S. Renshaw,[a,b] Patrick M. Slocombe,[c] Carl Doyle,[c] Alison Clargo,[c] Richard J. Taylor,[c] Christine E. Prosser,[a,c] Alistair J. Henry,[c] Martyn K. Robinson,[c] Lorna C. Waters,[a,b,2] Gill Holdsworth,[c,2] Mark D. Carr[a,b,2]

From the [a]Leicester Institute of Structural and Chemical Biology, and [b]Department of Molecular and Cell Biology, University of Leicester, Lancaster Road, Leicester, LE1 7HB, UK
[c]UCB Pharma, 208 Bath Road, Slough, SL1 3WE, UK

Running title: ***Structure, Dynamics and Functional Complexes of Dkk4***

Supporting Information Contents

1. Table S1. NMR constraints and structural statistics for the Dkk4$_N$ domain.
2. Figure S1. Characterisation of Refolded Dkk4$_{FL}$ and Dkk2$_{FL}$ Produced in *E. coli.*
3. Figure S2. Concentration Dependent Inhibition of Wnt Signalling by Dkk1 and Dkk4.
4. Figure S3. Amino Acid Sequence Conservation Between Mammalian and Reptilian Dkk4$_N$.
5. Figure S4. Representative Biolayer Interferometry Sensorgrams and Steady-State Analysis Curves Obtained for Dkk4$_{FL}$ and Dkk4$_N$ Binding to LRP6 E1E2-Fc
6. Figure S5. Binding of Dkk Proteins to Krm1 ECD-Fc and Characterisation of Krm1 ECD
7. Figure S6. Synergy between Krm1 and Dkk1 in HEK293 Tcf-Luc assay
8. Materials and Methods

NMR Constraints and Structural Statistics for $Dkk4_N$		
**a) No. of Constraints used in Final Structural Calculation**		
Intraresidue NOEs	223	
Sequential NOEs (*i*, *i*+1)	169	
Medium-range NOEs (*i*, *i*≤4)	66	
Long-range NOEs (*i*, *i* ≥5)	148	
Torsion angles	90	(46ϕ and 44Ψ)
Hydrogen bond	24	
Disulphide bond	30	
**b) Maximum and Total Constraint Violations in 70 Converged $Dkk4_N$ Structures**		
Upper distance limits (Å)	0.05 ± 0.06	0.1 ± 0.1
Lower distance limits (Å)	0.01 ± 0.01	0.0 ± 0.0
van der Waals contacts (Å)	0.16 ± 0.01	0.6 ± 0.2
Torsion angle ranges (°)	2.19 ± 0.27	2.6 ± 0.4
Average CYANA target function (Å^2)	0.11 ± 0.04	
**c) Structural Statistics for the Family of Converged $Dkk4_N$ Structures**		
Residues within allowed region of Ramachandran plot (%)		99.4%
Backbone atom RMSD to the mean structure (residues 44-47, 50-96 of $Dkk4_N$)		0.90 ± 0.23 Å
Heavy atom RMSD to the mean structure (residues 44-47, 50-96 of $Dkk4_N$)		1.40 ± 0.22 Å

Table S1. NMR constraints and structural statistics for the $Dkk4_N$ domain.

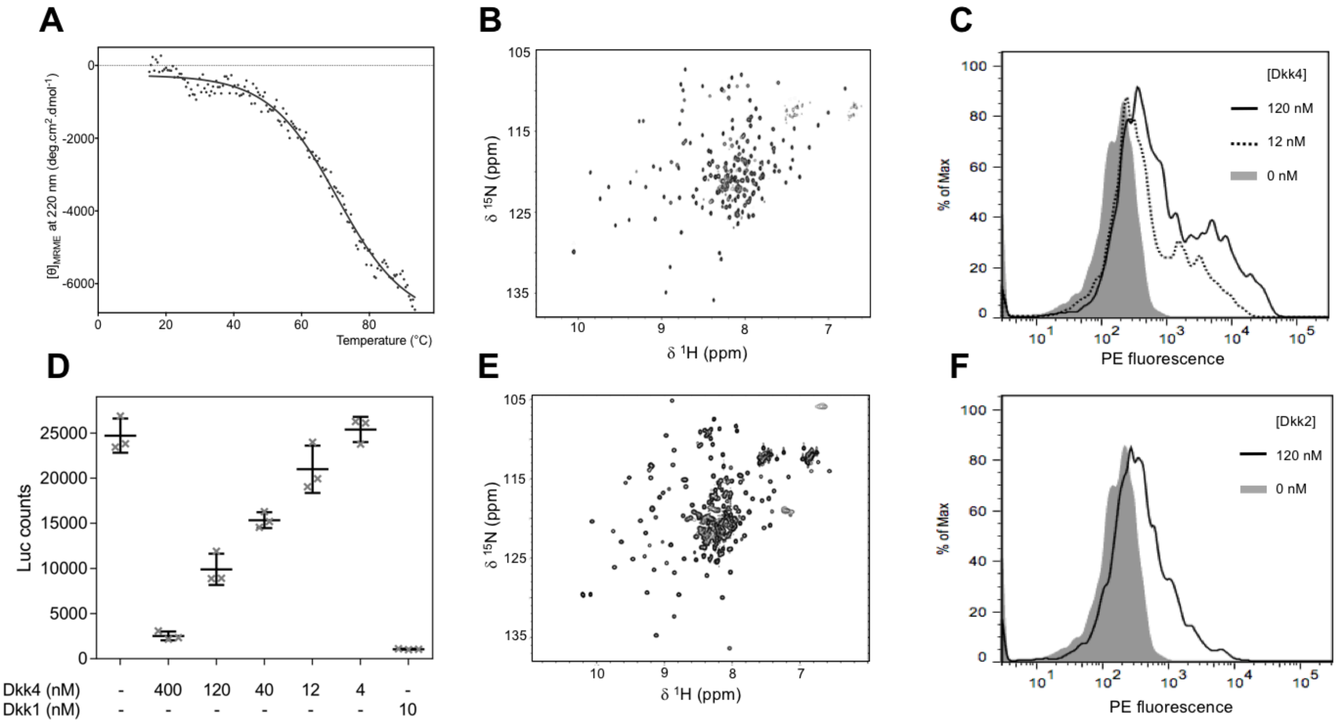

Figure S1. Characterisation of Refolded $Dkk4_{FL}$ and $Dkk2_{FL}$ Produced in *E. coli*

(A) Thermal stability of Dkk4 between 15 and 95 °C monitored by the change in CD signal intensity at 220 nm.

(B) $^{15}N/^{1}H$ TROSY spectrum of $^{15}N/^{13}C/^{2}H$-labelled $Dkk4_{FL}$ at pH 6.5 and 308 K.

(C) FACS analysis of the binding of His-tagged $Dkk4_{FL}$ to LRP6-transfected HEK293 cells (stained with anti-His PE antibody).

(D) Scatter plot of Wnt reporter assay illustrating dose-dependent inhibition of Wnt3a signalling by $Dkk4_{FL}$. HEK293 Tcf-Luc cells were stimulated with Wnt3a conditioned media for 24 hrs in the presence of the indicated concentrations of refolded recombinant $Dkk4_{FL}$, or recombinant Dkk1. The results are presented as a scatter plot showing individual data points, with bars indicating mean ± standard deviation.

E) $^{15}N/^{1}H$ TROSY spectrum of ^{15}N-labelled $Dkk2_{FL}$ at pH 5.5 and 308 K.

F) FACS analysis of the binding of His-tagged $Dkk2_{FL}$ to LRP6-transfected HEK293 cells (stained with anti-His PE antibody). This analysis was performed as part of the same experiment as the data shown in S1C and therefore uses the same negative (0 nM Dkk) control.

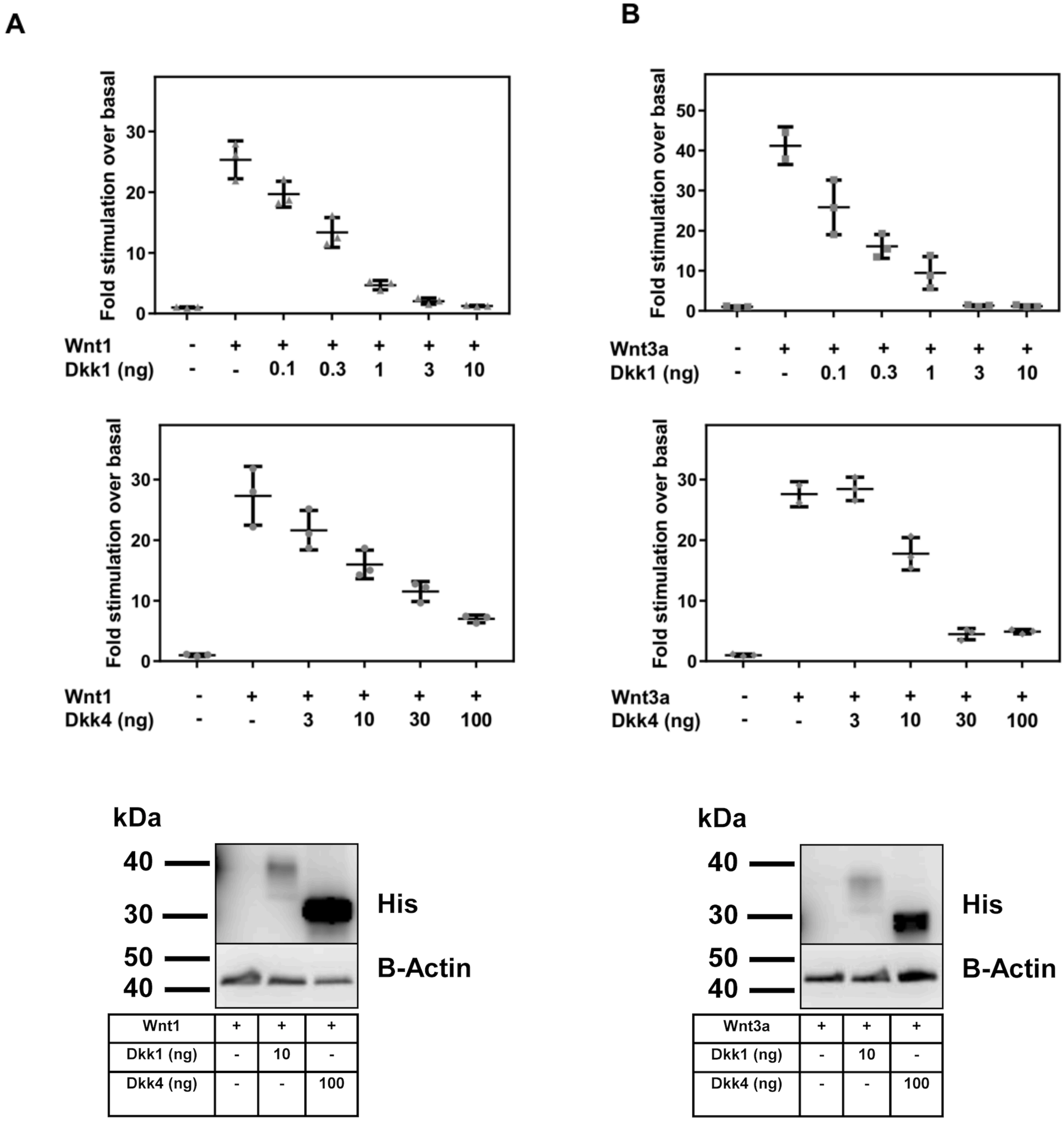

Figure S2. Concentration Dependent Inhibition of Wnt Signalling by Dkk1 and Dkk4.
Scatter plots of Wnt reporter assays illustrating concentration dependent inhibition of Wnt signalling by Dkk1 and Dkk4. HEK293 Tcf-Luc cells were transiently co-transfected with plasmids to express Wnt1 (A) or Wnt3a (B), together with an amount of plasmid encoding increasing amounts of Dkk1 (upper panel) or Dkk4 (middle panel). The results are presented as scatter plots showing individual data points, with bars indicating mean ± standard deviation. The relative expression of His-tagged Dkk proteins is shown in the Western blots below. Error bars show ± SD.

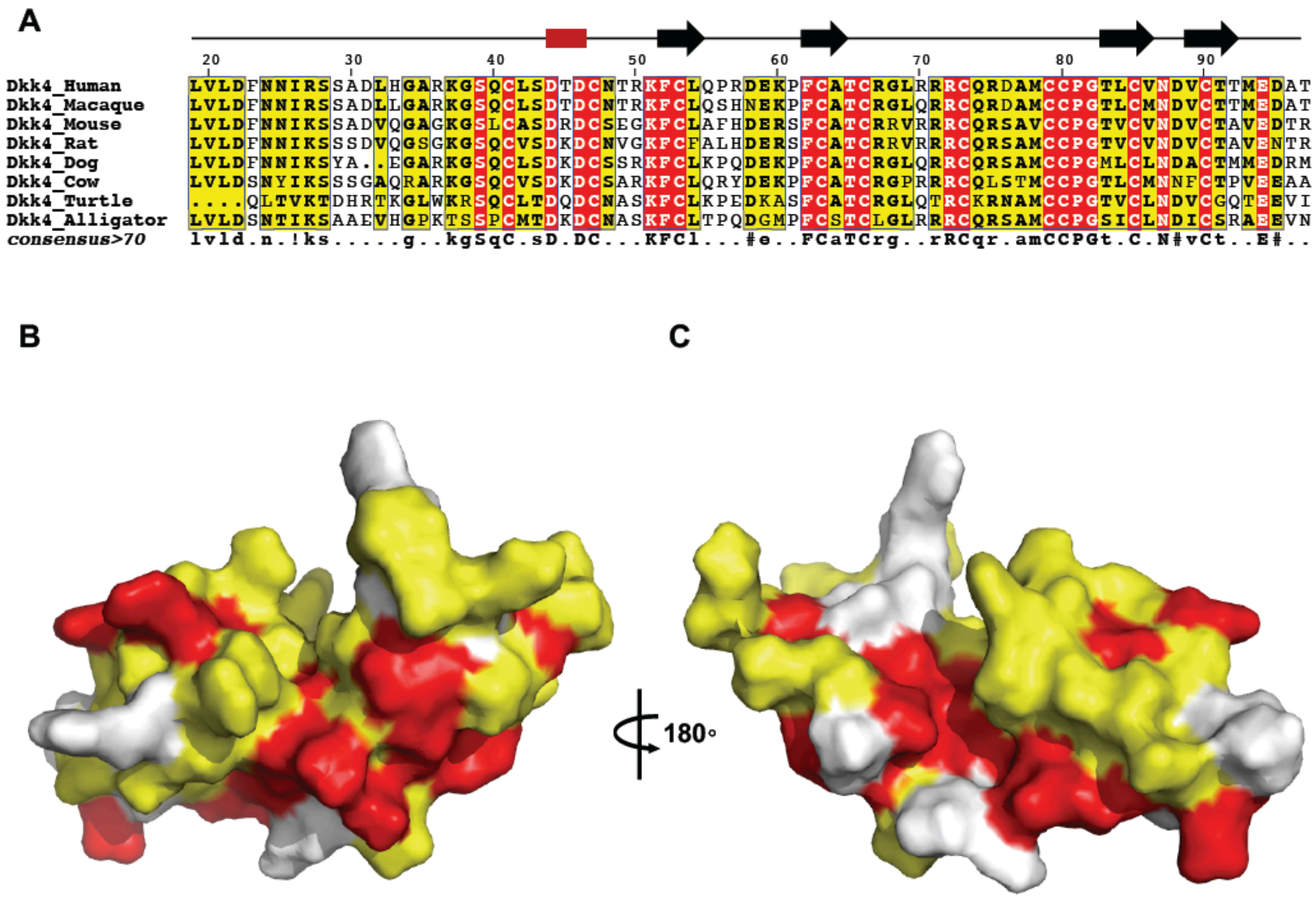

Figure S3. Amino Acid Sequence Conservation Between Mammalian and Reptilian Dkk4$_N$.

(A) Multiple sequence alignment of mammalian and reptilian Dkk4$_N$ (human, macaque, mouse, rat, dog, cow, turtle and alligator). Residues with completely conserved sequence identity are highlighted in red and those with conserved sequence similarity in 70% or more sequences are highlighted in yellow. The consensus sequence is shown below. Amino acids with completely conserved sequence identity are shown in uppercase; those with conserved sequence identity in over 70% of the sequences are shown in lowercase. Similar residues were grouped as follows: AVILM, FYW, KRH, DE, STNQ, PG and C. The symbol '!' is used to denote either I or V, '%' denotes F or Y, and '#' denotes anyone of NDQE.

(B) and (C) surfaces views of Dkk4 CRD1, with residues highlighted on the basis of sequence conservation, with residues that are identical across all the representative Dkk4 species shown in red and those with conserved sequence identity in over 70% of the sequences shown in yellow. The structure in (C) has been rotated by 180° about the Y axis relative to (B).

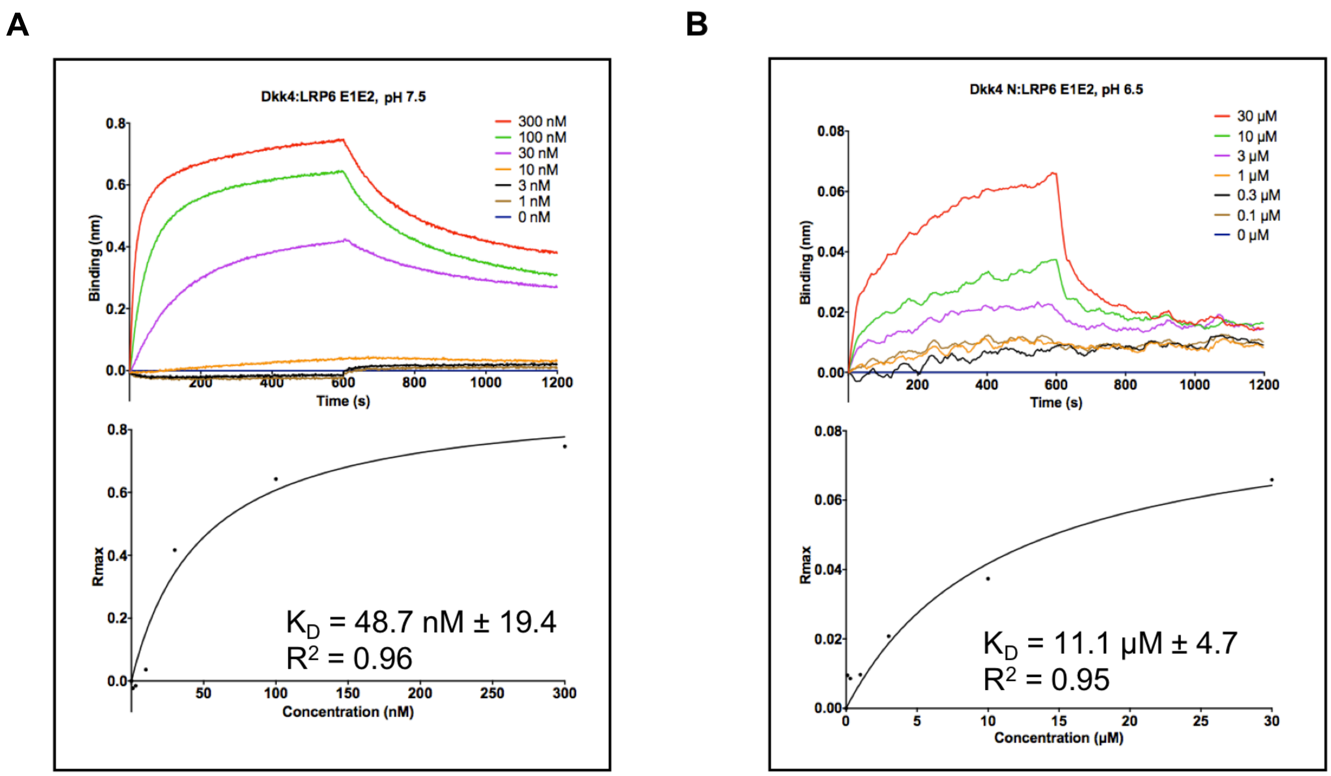

Figure S4. Representative Biolayer Interferometry Sensorgrams and Steady-State Analysis Curves Obtained for Dkk4$_{FL}$ and Dkk4$_{N}$ Binding to LRP6 E1E2-Fc

(A)-(B) The Dkk protein used, the binding partner and pH are indicated in the title of both panels. In each, the normalised sensorgrams (top) observed for the association and dissociation phases of the binding and the steady-state equilibrium binding curve (bottom) are shown. The steady-state curves obtained were fitted to a single binding model using Prism 6.0 to determine the K_Ds reported.

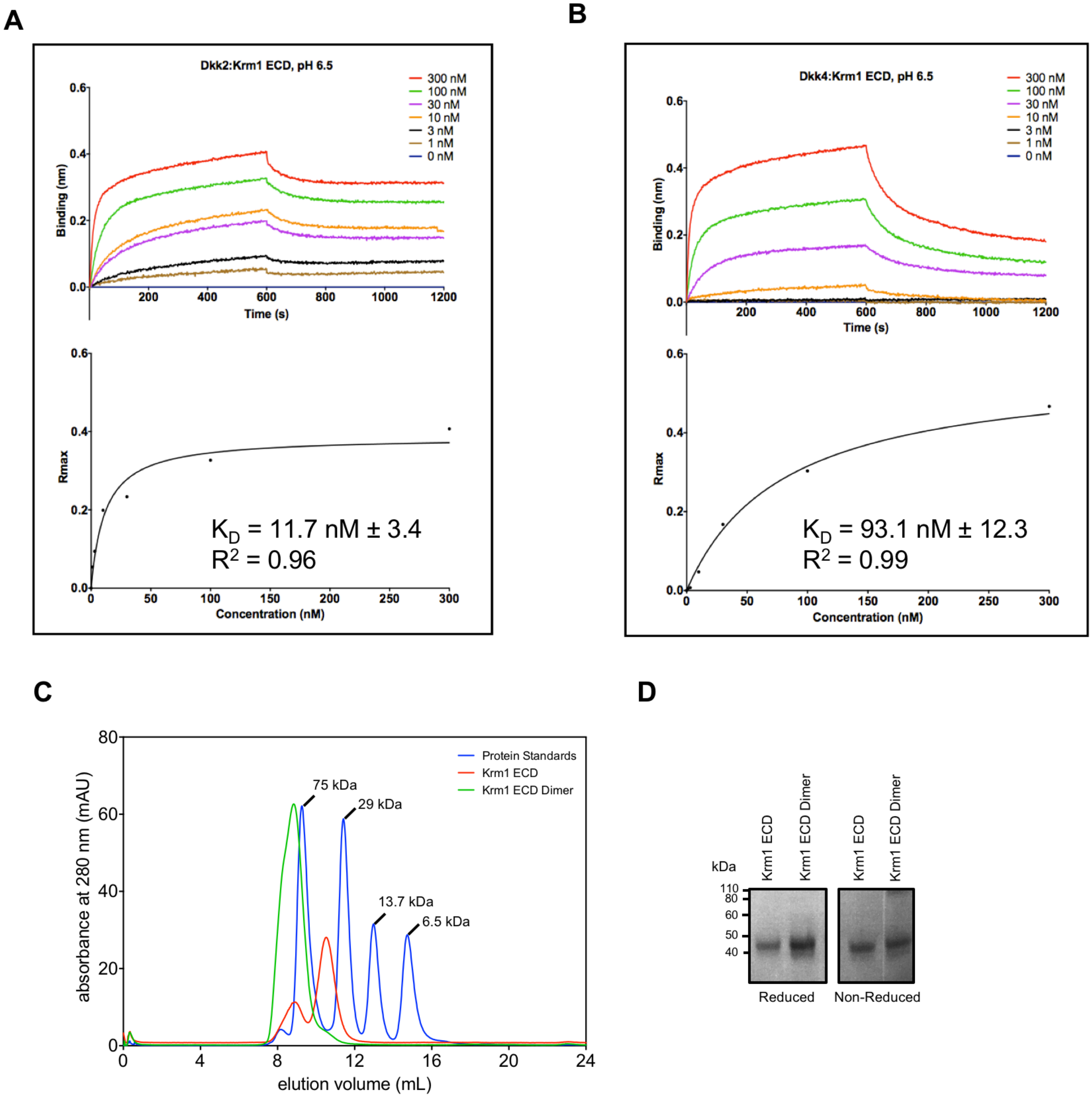

Figure S5. Binding of Dkk Proteins to Krm1 ECD-Fc and Characterisation of the Krm1 ECD

(A) and (B) Representative biolayer interferometry sensorgrams and steady-state analysis curves shown for $Dkk2_{FL}$ and $Dkk4_{FL}$ binding to Krm1 ECD-Fc. The Dkk protein investigated and pH are indicated in the title of each panel. In each the normalised sensorgram (top) observed for the association and dissociation phases of the binding and the steady-state equilibrium binding curve (bottom) are shown. The steady-state curves were fitted to a single binding model using Prism 6.0 to determine the K_Ds reported.

(C) Analytical size exclusion chromatography of purified fractions of untagged Krm1 ECD on a superdex 75 10/30 GL column, illustrating the dimerisation of purified monomeric Krm1 ECD (35 kDa) over a period of 1 month (red) whilst the purified Krm1 ECD dimer (70 kDa) remains stable (green). Protein standards (blue) are labelled with their corresponding molecular weights.

(D) SDS-PAGE gels of Krm1 ECD samples prior to analytical size exclusion chromatography analysis.

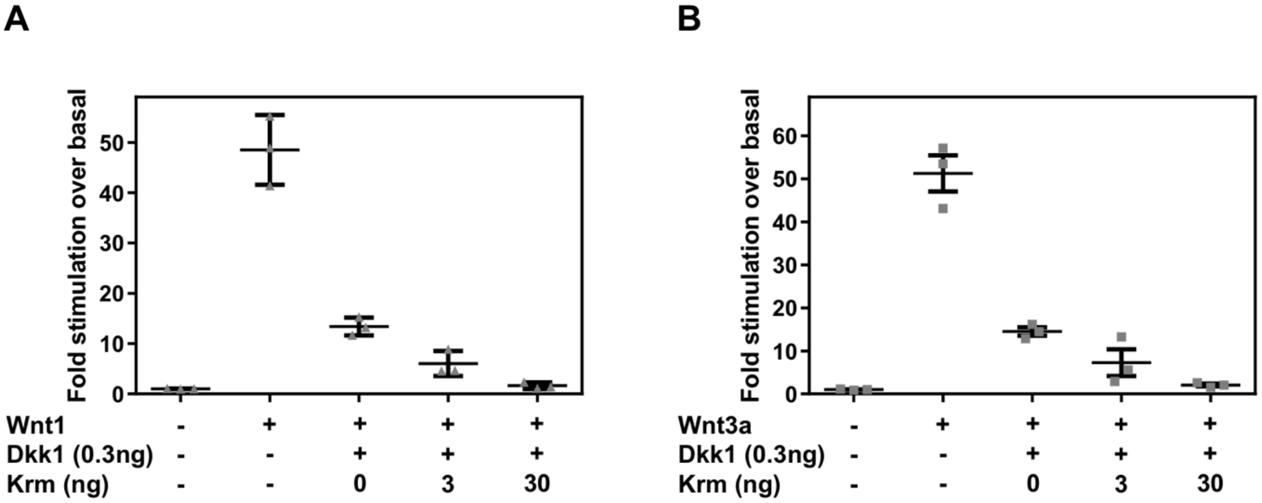

Figure S6. Synergy between Krm1 and Dkk1 in HEK293 Tcf-Luc assay
Scatter plots of Wnt reporter assays illustrating synergistic inhibition of Wnt dependent signalling by Dkk1 and Krm1.HEK293 Tcf-Luc cells were transiently co-transfected with plasmids to express (A) Wnt1 or (B) Wnt3a together with an amount of a plasmid encoding Dkk1 determined to give partial inhibition of Wnt-dependent signalling and increasing amounts of a Krm1 expressing plasmid. The results are presented as scatter plots showing individual data points, with bars indicating mean ± standard deviation.

Supporting Information Materials and Methods

Bacterial Expression Vectors

Genes encoding Dkk2$_{FL}$, Dkk4$_{FL}$ and Dkk4$_{N}$ (residues 38–259, 19–224 and 19–97 respectively; UniProtID Q9UBU2 and Q9UBT3) were cloned into a locally engineered ampicillin resistant pLEICS-05 plasmid, with a TEV protease cleavable C-terminal His$_6$ tag, which is based on pET-21a. Due to difficulties in expressing deuterated Dkk4$_{FL}$ using the pLEICS-05 construct the cassette encoding Dkk4$_{FL}$, the linker and C-terminal His$_6$ tag was codon optimised and inserted into the pET-29b(+) plasmid by GenScript.

Mammalian Expression Vectors

Constructs to express Dkk1$_{FL}$-His or Dkk4$_{FL}$-His were subcloned into a UCB proprietary expression vector following PCR amplification of the appropriate inserts from Origene ORF cDNA clones. A C-terminal His tag was engineered into the reverse primer for each insert. The Dkk1$_{FL}$-His construct used the native signal sequence whilst Dkk4$_{FL}$-His was cloned in-frame and downstream of the human VL signal sequence.

Sequences encoding Krm1 ECD (residues 20-326 and UniProtID Q96MU8-1 with the isoform 2 and 3 insertion at position 31 of E to GPE) and Krm2 ECD (residues 26-332 and UniProt ID Q8NCW0-1) were cloned into the pNAFH-hFc vector, which contained a TEV cleavable C-terminal human Fc tag.

LRP6-E1E2 consisting of the first two propeller and EGF domains (residues 1-637, UniprotID O75581) with a C-terminal TEV protease site was isolated by PCR and cloned into the pMH vector, which contained an in-frame human Fc expression tag. All plasmid inserts were verified by DNA sequencing prior to transfection (Holdsworth et al., 2012).

Preparation of Dkk Proteins

For the production of unlabelled, ^{15}N, or $^{15}N/^{13}C$ labelled proteins a single *E. coli* Rosetta™ 2(DE3) colony transformed with pLEICS-05 encoding Dkk2$_{FL}$, Dkk4$_{FL}$ or Dkk4$_{N}$ was grown in LB or minimal media (containing 1 g/L ^{15}N ammonium sulphate and 3 g/l ^{13}C glucose if required, as the sole nitrogen and carbon sources) at 37 °C to an OD_{600} of 0.6-0.8. Protein expression was induced by the addition of 0.4 mM IPTG and cultures incubated at 37 °C for 4 hrs. For $^{15}N/^{2}H$ or $^{15}N/^{13}C/^{2}H$ labelled Dkk4$_{FL}$ expression a single *E. coli* BL21 (DE3) colony was transformed with pET29-b(+) encoding codon-optimised Dkk4$_{FL}$. Cells were grown in $^{15}N/^{2}H$ or $^{15}N/^{13}C/^{2}H$ minimal media (100% D_20) at 37 °C to an OD_{600} of 0.8-1.0. Protein expression was induced by the addition of 0.4 mM IPTG and cultures incubated overnight at 18 °C.

Cell pellets containing insoluble expressed Dkk proteins were resuspended in lysis buffer (50 mM Tris, 2 mM EDTA, 0.1 % (v/v) Triton X-100, pH 8.0) and lysed using a French® Pressure Cell Press (Thermo Fisher Scientific) at 1000 psi with the 40 000 max psi French® Pressure Cell (Thermo Fisher Scientific) pre-chilled to 4 °C. To isolate the inclusion bodies the cell lysate was centrifuged (12000 x g for 20 mins at 4 °C) and the inclusion bodies were washed 2-3 times with wash buffer (50 mM Tris, 10 mM EDTA, 0.5 % (v/v) Triton X-100, 10 mM DTT, pH 8.65) using a glass homogeniser on ice. The homogenate was centrifuged (12000 x g for 15 mins at 4 °C) after each wash step. After the final wash step the pellet was resuspended in 20 mL resolubilisation buffer (50 mM Tris, 11 mM DTT, 5 M GdmHCl, pH 8.70) before being centrifuged (12000 x g for 20 mins at 4 °C). The supernatant was decanted and the protein concentration determined from the A_{280}.

The resolubilised material was diluted to a final concentration of 2 mg/mL in resolubilisation buffer. This was diluted 100-fold by adding drop-wise to room temperature refolding buffer (50 mM Tris, 0.45 M GdmHCl, 0.78 mM GSH, 0.44 mM GSSG, pH 8.65), which was gently stirred. Once all the resolubilised material had been added to the refolding buffer it was stirred for a further 2 hrs before transferring to 4 °C where it was left unstirred overnight. Refolded material was filtered and concentrated to a volume of 50-100 mL using a Vivaflow 200 polyethersulfone (PES) membrane (5000 or 10 000 MWCO; Sartorius) attached to MasterFlex easy-load pump (Cole Parmer).

The refold mix was dialysed against 50 mM Tris, 100 mM NaCl, pH 7.5 and applied to a 5 mL NiNTA superflow cartridge (QIAGEN) pre-equilibrated with 10 column volumes (CV) of loading buffer (50 mM Tris, 100 mM NaCl, 20 mM imidazole, pH 7.5). The column was washed with 25 CV loading buffer and the bound Dkk proteins were eluted using a linear gradient of loading buffer supplemented with 500 mM imidazole over 10 CV. Fractions containing Dkk were pooled and dialysed against size exclusion chromatography (SEC) buffer (25

mM bis-Tris, 100 mM NaCl, pH 5.5). The dialysed protein solution was concentrated to 5-10 mL using an Amicon-Ultra15 centrifugal filter unit with a regenerated cellulose membrane (10 or 3 kDa NWCO; Merck Millipore).

SEC was performed using a pre-packed HiLoad™ 16/60 Superdex™ 75 prep grade column (GE Healthcare) at room temperature. The column was pre-equilibrated with 3 CV of SEC buffer prior to loading 5 mL of protein onto the column at a rate of 1 mL/min. The A_{280} was monitored throughout the run and 2 mL fractions were collected and analysed by SDS-PAGE. Fractions from under each elution peak were pooled separately and stored at 4 °C. A calibration was performed for the SEC column to allow estimations of molecular weights. The calibration was done using the Gel Filtration LMW Calibration Kit (GE Healthcare). Globular protein standards were made up in 50 mM Na_2HPO_4, 100 mM NaCl, pH 7.5 at the manufacturers recommended concentrations. A linear regression analysis was performed on the relationship between the logarithm of the molecular weight and elution volume for the globular protein standards and was used to calculate the apparent molecular weights of the species in all subsequent samples analysed by size exclusion chromatography.

Preparation of Krm Proteins

For the production of Krm ECD proteins 2 x10^8 CHO-SXE cells were resuspended in 10 mL Earle's balanced salt solution (EBSS) and 4 mg of recombinant plasmid DNA was added. The cells were transfected using the Gene Pulser Xcell™ system (BioRad) by adding 800 µL of the plasmid DNA/cell mixture into a Gene Pulser electroporation cuvette and applying ~280-300 V. This was added to 1 L CD Gibco® CHO medium (supplemented with 0.01 % (v/v) GlutaMAX™ supplement and 0.002 % (v/v) Gibco® Antibiotic Antimycotic), which was pre-warmed to 37 °C in a water bath. Cultures were incubated at 37 °C with shaking overnight following which the temperature was reduced to 32 °C. Sodium butyrate (3 mM) was added on day 3. On day 7, the cell number and viability were checked.

The cultures were harvested by centrifugation (4000 x g for 30 mins at 4 °C) and the supernatant filtered using a Sartobran® P 0.2 µm filter (Sartorius) and a Millipak® 40 Gamma Gold 0.22 µm filter (Merck Millipore). A final concentration of 0.02 % (w/v) NaN_3 was added to prevent microbial growth. For purification, 10 mL of MabSelect SuRe Protein A beads (GE Healthcare) were pre-equilibrated with 5 CV protein A purification buffer (50 mM Na_2HPO_4, 100 mM NaCl, pH 7.5) at room temperature. The filtered supernatant was loaded onto the medium at 3 mL/min and the A_{280} and conductivity measured throughout the run. The beads were subsequently washed with 3 CV protein A purification buffer and resuspended in 10 mL protein A purification buffer.

The hFc tag was cleaved using TEV protease whilst the Krm recombinant protein was still bound to the protein A beads. 0.01 % (v/v) TEV protease (3 mg/mL; UCB Pharma) was added to the protein A beads and left at 4 °C overnight with gentle rocking. The beads were centrifuged (4500 x g for 5 mins at 4°C) and the supernatant decanted. The protein A beads were gently resuspended in 10 mL protein A purification buffer and washed to remove all cleaved Krm ECD recombinant protein. The supernatant was combined and concentrated to a final volume of 5-10 mL using an Amicon Ultra15 centrifugal filter unit with a regenerated cellulose membrane (10 kDa NWCO; Merck Millipore) prior to SEC. SEC was performed as described above using protein A purification buffer.

Preparation of LRP6 Proteins

Expression and purification of LRP6 E1E2 were carried out as described previously (Holdsworth et al., 2012).

Analysis of Purified Proteins

All proteins were analysed by SDS-PAGE and mass spectrometry under reducing and non-reducing conditions. To obtain an intact mass for $Dkk2_{FL}$, protein samples (100 µM) were submitted for liquid chromatography-mass spectrometry (LC-MS/MS) analysis. Experiments were conducted under non-reducing conditions using a LTQ Orbitrap spectrometer (Thermo Fisher Scientific). All other samples were analysed using electrospray ionisation mass spectrometry (ESI-MS) on an Acquity H-Class UPLC system with a Xevo G2 QTof detector.

References

Holdsworth, G., Slocombe, P., Doyle, C., Sweeney, B., Veverka, V., Le Riche, K., Franklin, R. J., Compson, J., Brookings, D., Turner, J., Kennedy, J., Garlish, R., Shi, J. Y., Newnham, L., McMillan, D., Muzylak, M., Carr, M. D., Henry, A. J., Ceska, T., and Robinson, M. K. (2012) Characterization of the Interaction of Sclerostin with the Low Density Lipoprotein Receptor-related Protein (LRP) Family of Wnt Co-receptors. *Journal of Biological Chemistry* **287**, 26464-26477
